# A Socio-Ecological Approach for Identifying and Contextualising Spatial Ecosystem-Based Adaptation Priorities at the Sub-National Level

**DOI:** 10.1371/journal.pone.0155235

**Published:** 2016-05-26

**Authors:** Amanda Bourne, Stephen Holness, Petra Holden, Sarshen Scorgie, Camila I. Donatti, Guy Midgley

**Affiliations:** 1 Conservation South Africa, Springbok, South Africa; 2 Centre for African Conservation Ecology, Department of Zoology, Nelson Mandela Metropolitan University, Summerstrand, Port Elizabeth, South Africa; 3 Conservation South Africa, Cape Town, South Africa; 4 Plant Conservation Unit, University of Cape Town, Cape Town, South Africa; 5 The Gordon and Betty Moore Center for Science, Conservation International, Arlington, VA, United States of America; 6 Global Change Biology Unit, Stellenbosch University, Stellenbosch, South Africa; University of Vermont, UNITED STATES

## Abstract

Climate change adds an additional layer of complexity to existing sustainable development and biodiversity conservation challenges. The impacts of global climate change are felt locally, and thus local governance structures will increasingly be responsible for preparedness and local responses. Ecosystem-based adaptation (EbA) options are gaining prominence as relevant climate change solutions. Local government officials seldom have an appropriate understanding of the role of ecosystem functioning in sustainable development goals, or access to relevant climate information. Thus the use of ecosystems in helping people adapt to climate change is limited partially by the lack of information on where ecosystems have the highest potential to do so. To begin overcoming this barrier, Conservation South Africa in partnership with local government developed a socio-ecological approach for identifying spatial EbA priorities at the sub-national level. Using GIS-based multi-criteria analysis and vegetation distribution models, the authors have spatially integrated relevant ecological and social information at a scale appropriate to inform local level political, administrative, and operational decision makers. This is the first systematic approach of which we are aware that highlights spatial priority areas for EbA implementation. Nodes of socio-ecological vulnerability are identified, and the inclusion of areas that provide ecosystem services and ecological resilience to future climate change is innovative. The purpose of this paper is to present and demonstrate a methodology for combining complex information into user-friendly spatial products for local level decision making on EbA. The authors focus on illustrating the kinds of products that can be generated from combining information in the suggested ways, and do not discuss the nuance of climate models nor present specific technical details of the model outputs here. Two representative case studies from rural South Africa demonstrate the replicability of this approach in rural and peri-urban areas of other developing and least developed countries around the world.

## Introduction

The impacts of climate change present a significant challenge to long-term economic development and human well-being [1,2]. It adds an additional layer of complexity to the already challenging goals of sustainable development and biodiversity conservation. Ecosystem-based solutions are a vital part of helping people adapt to climate change. Ecosystems provide flood control, coastal protection, fresh water, and sustainable food supplies under climate variability and change, including extreme events. The conservation, restoration, and sustainable management of ecosystems is therefore critical for protecting communities from the short-term detrimental impacts of climate change (such as increased storm intensities) and buffering against its long-term impacts (such as decreasing food security). Solutions that harness the potential of nature to deliver an adaptation benefit are widely known as ecosystem-based adaptation (EbA) [3,4].

EbA, defined by the Convention on Biological Diversity [5] as *the use of biodiversity and ecosystem services to help people adapt to the adverse effects of climate change as part of an overall adaptation strategy*, is generating growing interest globally as a realistic and desirable adaptation option, particularly for developing and least developed countries. For EbA to be effective it should be planned and implemented at the local level. This allows for a direct link to local contexts and place-specific challenges. Furthermore, it enables effective adaptive management, and the embedding of climate change into local planning and implementation, including into service delivery, land use and infrastructure planning, and natural resources management [6].

Poverty, inequality, weak governance, population growth, inadequate infrastructure, and poorly planned, market driven development interact with climatic forces to shape the direction of change in landscapes around the world [7,8]. At a local level, the distribution and level of access to resources, such as wealth, municipal services, infrastructure, education and natural resources, influences a community’s vulnerability to potential risks and hazards, including climate change. As rural and peri-urban populations rely more directly on ecosystem services than their urban counterparts, such interactions may lead to drastic changes in these landscapes [8].

Developing effective EbA responses essentially requires an integrated understanding of socio-ecological vulnerability and the ways in which ecosystems can assist local communities to adapt to climate change [3,9]. Local government officials seldom have access to information on climate change and ecosystem functioning [6, 10]. This is especially the case when considering the local level information required for obtaining an effective understanding of the potential contribution that ecosystems can make to climate change adaptation [11]. To support EbA planning and decision-making, a replicable approach for collating and presenting complex climatic and socio-ecological information in a simple format and at a comprehensible and relevant spatial scale is required.

The available EbA decision-support tools and guidance documents focus on i) generalised principles and best practice for EbA (e.g.[12]), ii) identifying EbA interventions and providing lists of examples for implementation (e.g. [13]), and iii) spatially identifying climate change and disaster risk impact hotspots to guide infrastructure investments (e.g.[14,15]). Many existing tools assume that the climate change information for a country is in an accessible and understandable format for providing the context that decision makers require for prioritising EbA interventions and related decision-making at the local level. These tools are insufficient for supporting local level planning and implementation as they lack comprehensive spatial planning components, are not packaged in a simple and understandable format, or are limited in their integration of social, ecological and climatic aspects [12 – 15].

The many studies assembling EbA case material and describing the benefits or effectiveness of EbA interventions are useful for obtaining high-level political buy-in, but lack context-specific information to guide local level decision-making, planning and implementation [4,16] and do not include information on where to implement or how to incorporate different ecosystems, local communities, and the social-ecological interactions between them. Work on the valuation, mapping, and prioritisation of ecosystems for ecosystem services provisioning [17–19] is prolific and mapping of household level ecosystem service use [20,21] is a growing practice in the socio-ecological systems literature. For example, a recent paper by Hamann et al [21] made use of the South African census data alongside other national spatial datasets to test underlying factors that influence the distribution of socio-ecological systems and presented the approach for replication in other regions. They identify low, medium, and high levels of direct ecosystem service use among households in the country, related to green and red loop ecosystem service systems described by Cumming et al [22]. Applying these GIS-based analyses to spatially represent relationships between socio-economic ecosystem services data, as presented in Hamann et al [21], is useful for providing a spatial perspective of the ability of ecosystems to support human well-being. However, the approach does not explicitly consider the current and future impacts of climate change or the contributions ecosystems can make to local adaptation efforts.

Therefore, current approaches for supporting EbA planning and implementation do not focus on the spatial integration of climate, social and ecological aspects. Furthermore, they do not use country-driven administrative units to spatially contextualise results and to ensure their direct relevance for local level political, administrative and operational decision makers. Particularly, there is a limited understanding of how to effectively incorporate climate change projections into EbA tools for local level decision-making. This paper presents a novel and systematic approach to address the gaps in existing EbA decision support tools and guidance documents. The approach is innovative as it aligns nodes of socio-ecological vulnerability with areas of high ecological climate resilience and ecosystem service provisioning to identify potential locations for the implementation of EbA at a local level. A spatial planning approach drawing on conservation planning techniques (see [23]) was developed for i) mapping local EbA priorities in rural and peri-urban areas in developing and least developed countries using critical social and ecological indicators of vulnerability and resilience; and ii) contextualising climate change projections and impacts to assist decision makers with prioritising locations for the on-the-ground implementation of EbA interventions. We argue that mainstreaming of climate change and adaptation options at the sub-national level [24–26] is greatly enhanced by a taking a spatial and scenario based planning approach at the scale of the known and familiar administrative boundary. This is so even though social and ecological processes routinely transcend these boundaries.

We present the results of this approach as applied to two representative case studies in South Africa, one in an arid system (Namakwa District Municipality—NDM) and one in a more mesic system (Alfred Nzo District Municipality—ANDM). Together these districts represent six of South Africa’s eight major biomes. The final products of the approach comprise maps showing i) predicted biome changes in response to climate change scenarios, and ii) EbA priority areas that incorporate both social and ecological vulnerability. The maps address climate change, the environment, and local government priorities for human development and poverty alleviation. They systematically incorporate socio-economic priorities for local governments into spatial EbA priority areas, providing a means of prioritising location for EbA implementation to maximise climate adaptation benefits where resources are limited. This spatial planning approach has been adopted in both NDM and ANDM, playing a substantial role in local level planning. It can be replicated in other areas and at larger scales. Together, these products, enable EbA implementation in priority areas that align with the delivery of other social benefits [27,28]. Rather than emphasising the specific technical outputs of the local models and analyses, the purpose of this paper is to demonstrate a methodology for combining information into user-friendly spatial products for local level decision making on EbA, illustrated through the use of case studies.

## Methods

### Overview

We used two GIS-based approaches to develop spatial products that communicate i) climate change projections and impacts, and ii) EbA priorities to local decision makers. The first product, a set of ‘biome stability’ maps, is intended for building local capacity to understand future climate change projections and impacts, work with a range of plausible climate futures, and use climate projections to inform planning discussions. Climate impacts information is contextualised and presented at a familiar scale, that of the local administrative boundary. The biome stability maps, essentially the outputs of climate envelope models, highlight natural systems that are likely to be more or less stable at a broad scale under changing climate conditions. The second product is for guiding on-the-ground EbA implementation. These novel ‘EbA priority areas’ maps identify high priority sites for EbA implementation and can be replicated in developing and least developed countries using socio-ecological indicators of vulnerability and resilience at the scale of the relevant sub-national administrative unit responsible for land use planning and service delivery in a country. The EbA priority areas maps are designed to enable decision-makers to visualise priority sites for fine scale local level planning for EbA implementation. Maps do not account for larger scale ecological processes, although the authors acknowledge that these are important. Both spatial products developed are explicitly targeted at the scale at which local land use planning decisions are made. For the two South African case studies presented here, this level of governance is the District municipality.

### Biome stability maps

A maximum entropy model, MaxEnt [29–31] (the industry standard software for habitat modelling), was used to develop a biome distribution model in order to predict the potential future distribution of biomes in the NDM (Succulent Karoo, Desert, Nama Karoo, and Fynbos [32]) and the ANDM (Grassland and Savanna [32], with some coastal belt forest patches) based on climatic variables. MaxEnt model outputs for current, medium and longer-term biome distribution were overlaid to estimate biome stability. Following Araujo and Townsend Petersen [33], we apply the term climate envelopes to refer to the estimation of multivariate climatic variables best matching observed biome distribution.

The present day climate values used for this analysis are based on daily agrohydrological data for rainfall and temperature for the period 1960–1999 [34–36]. The climate scenario data used were based on the difference between future and current values for each of three Global Climate Models (i.e. future predicted value–control values produced by the model for present conditions), dynamically downscaled to Southern Africa, to represent three plausible scenarios of temperature and rainfall change over the medium and longer term. The models are MIROC3.2-medres [37], ECHAM5 [38,39], and CSIRO 3.5 [40], downscaled using CCAM [41] applying a relatively high end, or ‘unmitigated’ emissions scenario–A2 SRES [41,42]. Outputs for the medium term (2050) correlate to models for the period 2040–2059, and for the longer term (2100) to models for the period 2081–2100. Although not included in the analysis, the authors also considered the climate modelling outputs of South Africa’s Long Term Adaptation Scenarios (LTAS) Flagship Research Programme. Specifically, this included drawing on the Climate Trends and Scenarios work [43], which presents statistically and dynamically downscaled climate projections for South Africa based on ten Global Climate Models and four emissions scenarios (SRES B1, SRES A2, RCP 4.5, and RCP 8.5). The model outputs of this analysis were compared to the LTAS model outputs, which represent a fuller range of plausible climate futures.

The ability of the MaxEnt model to predict the future potential distributions of biomes was tested by using it to model the current distribution of biomes. The model was 86.02% accurate at modelling the current distribution of biomes, producing a map that closely correlated with the actual distribution of biomes.

### EbA priority areas maps

**Fig 1:**
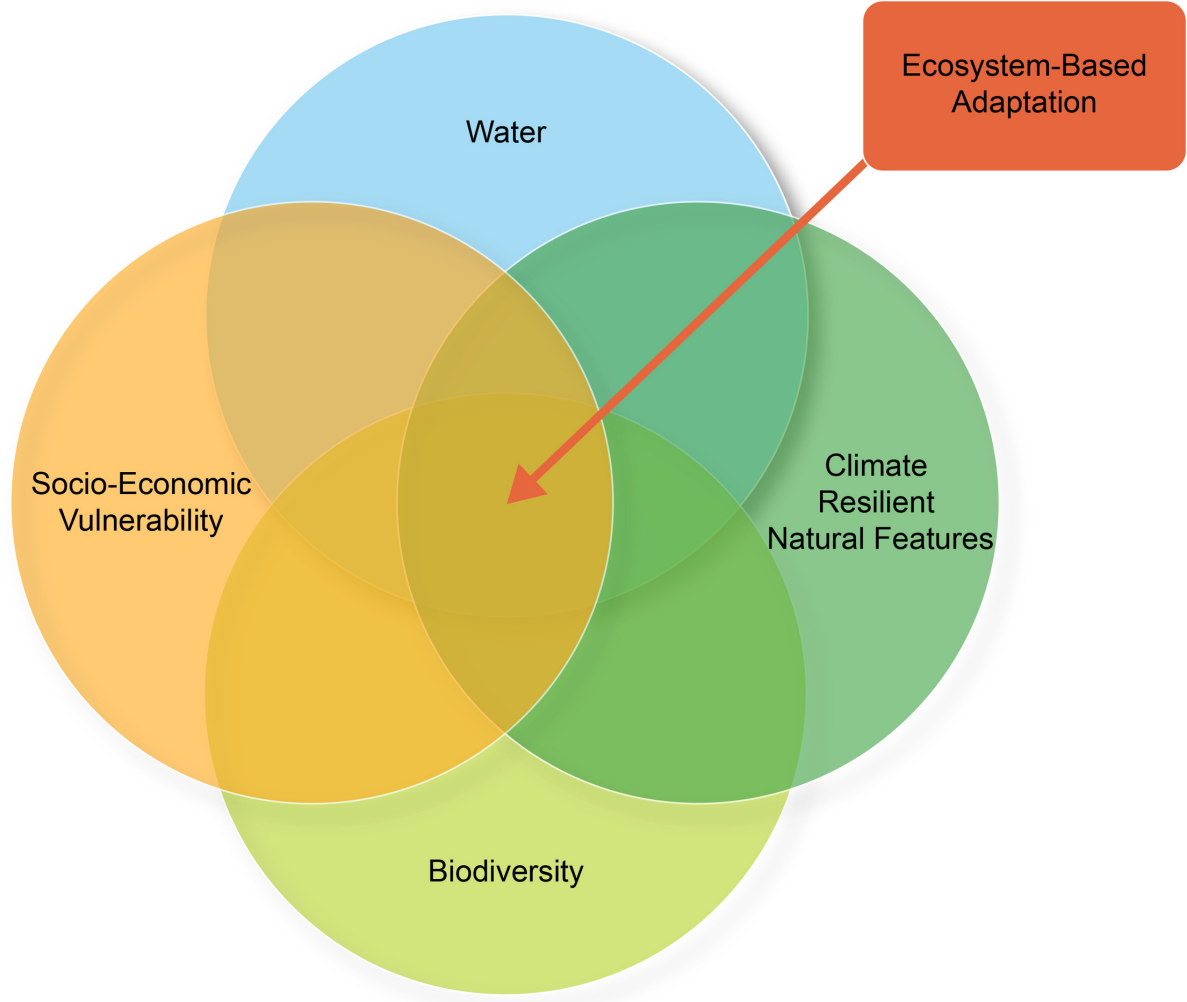
Summary of the analysis and integration process. Reprinted from [44] under a CC BY license, with permission from CSA, original copyright 2015.

EbA priority areas maps are made up of a set of composite maps for i) water ecological infrastructure, ii) important biodiversity (underpinning a range of ecosystem services), iii) natural features supporting an ecosystem’s ability to adapt to climate change, and iv) nodes of socio-economic vulnerability and high social demand for natural resources and ecosystem services. Fig 1 summarises the analysis and integration method. EbA priority areas lie at the intersection of all four categories i.e. where water, biodiversity, climate resilient natural features, and social needs significantly overlap. The overlapping areas highlight where natural features are likely to provide resilience to the impacts of climate change. They also identify locations where vulnerable communities of people are currently directly dependent on these natural features for their livelihoods, as well as likely to need them to support their ability to adapt to a changing climate in the future.

**Fig 2:**
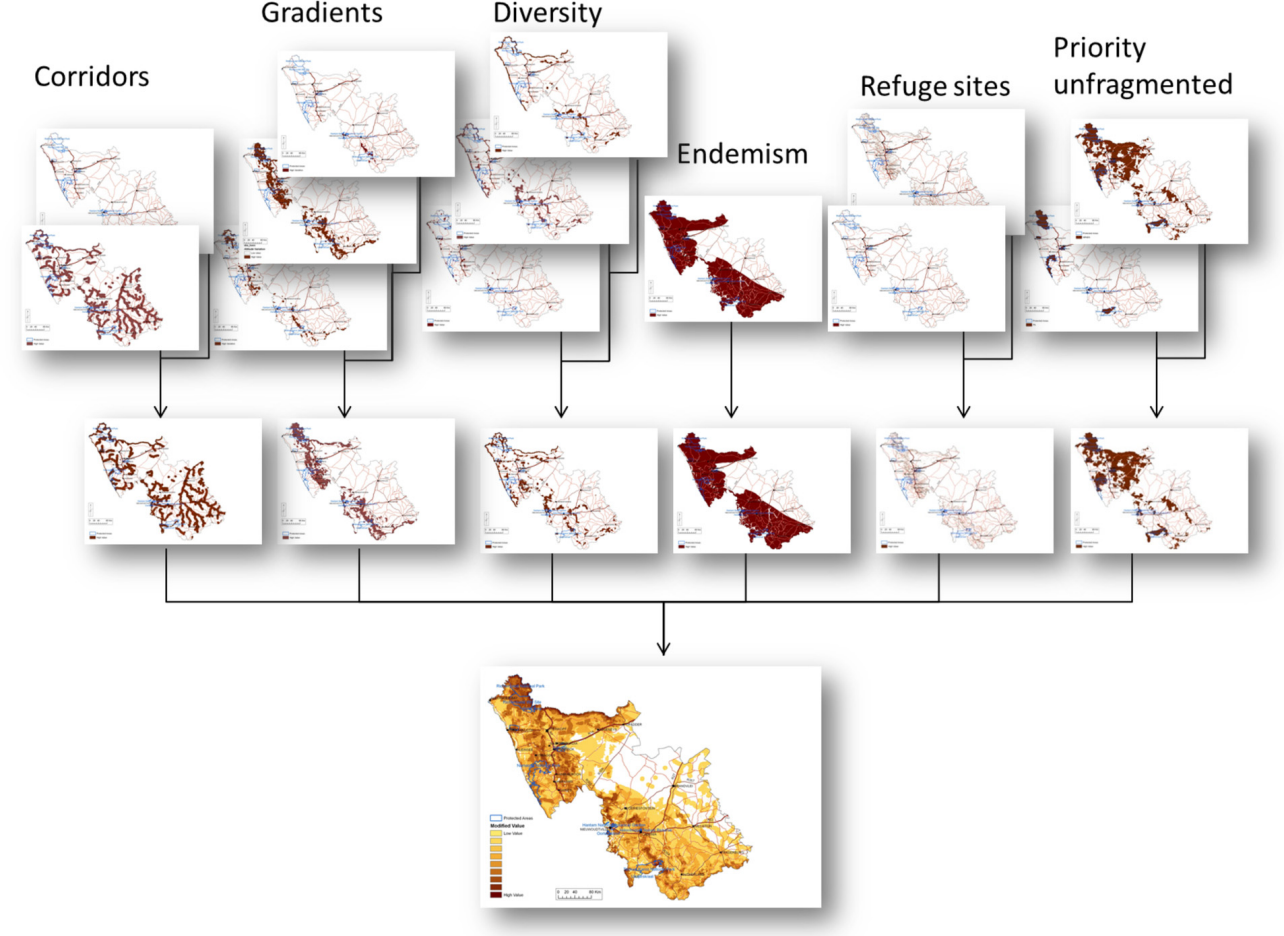
Diagram illustrating the integration method used to develop composite maps for each category of information making up the final EbA priority areas maps. This particular example shows all the individual layers which made up the composite map for natural features supporting resilience to climate change impacts at a landscape scale in the Namakwa District Municipality, South Africa. Specific spatial data on corridors, gradients, diversity, endemism, refugia, and unfragmented landscapes were overlaid to produce composite maps for each category of natural feature with the potential to contribute to climate change adaptation. These were then overlaid to produce a single map, at the District municipality scale, which summarises all the climate resilient natural features identified, showing in the darker areas where there are high levels of overlap (i.e. where many climate resilient natural features are present). Reprinted from [44,45] under a CC BY license, with permission from CSA, original copyright 2015.

Each of the four categories of information used in the EbA priority areas maps were first developed separately, as a set of composite maps for water, biodiversity, climate resilient natural features, and social needs. In other words, each individual composite map is composed of several layers of features and presented as a separate spatial product that can be used on its own for decision-making on, for example, water resources management, or protected areas expansion. Fig 2 provides a detailed example of the integration method used for all of the composite maps, following a standard multi-criteria GIS analysis and integration approach. The same method was used for developing spatial decision-making products for all four categories described above and for their final integration into the EbA priority areas maps.

Although the four categories of information that make up an EbA priority area are generalisable, the specific layers which populate each category were determined locally, by the local context as well as by the available data. In South Africa, available water data included areas important for water production and water quality, erosion control, and flood attenuation. The biodiversity information available in South Africa included biodiversity priorities, critical biodiversity areas, and protected area expansion priorities. For climate resilient natural features, the information available in South Africa included coastal and riparian corridors, as well as temperature, rainfall and altitudinal gradients, and centres of endemism.

Finally, the distribution and the level of access to resources, such as wealth, municipal services, infrastructure, education and natural resources, influences a community’s vulnerability to potential risks and hazards. Hotspots of socio economic vulnerability to climate change include those areas where poverty levels are high and where people are most directly dependent on the environment for the delivery of ecosystem services. Determining spatially the areas important for adaptation action in terms of social and economic features involved mapping:

1. Population densities and locations of households. Data on the location of individual dwellings was used to provide an accurate representation of population density at a fine scale, with the location of buildings being used as a proxy for population distribution. This approach was necessary as the available census data summarised data for large areas.
2. Levels of poverty were initially assessed based on the proportion of households with an annual income below the poverty line (in South Africa this is ZAR9,600 per annum, the value of one average government social grant per household, and roughly equivalent to USD700). We then developed a dependency ratio of employed people to unemployed people, discouraged work seekers, people who are not economically active, and those under the legal working age. We also developed an index of poverty. This included an analysis of access to services such as access to sanitation, piped water within 200m, refuse collection, and electricity for lighting, and levels of consumption measured by ownership of a range of goods including a car, cell phone, computer, DVD player, refrigerator, radio, satellite television, stove, television, vacuum cleaner and washing machine.
3. A specific environmental dependency index capturing levels of local community direct dependence on locally defined critical ecosystem services. In the arid zone case study these were freshwater derived from underground aquifers and access to communal grazing resources, while in the more mesic case study these were natural building materials, freshwater from surface sources such as rivers and streams, and access to wood for fuel.

Socio-economic indicators were combined with national land cover maps in order to account for the quality of ecosystem services provided by natural and modified areas. Transformed areas were assumed not to provide high value ecosystem services and were removed from the analysis. The inclusion of the land cover layer highlighted where areas of high natural resource dependence occurred in conjunction with natural or semi-natural (modified by still maintaining ecological function) areas critical to the supply of ecosystem services [46]. This produced a composite map of social demand on the natural environment. All variables considered were equally weighted. The final integrated map reflects climate vulnerability hotspots in natural and semi natural areas based on where people rely directly and heavily on ecosystem services and where general poverty is high.

Table 1 lists each natural and socio-economic feature included in the EbA priority areas maps for the two case studies, as well as the databases and data sources used. The references listed under the table relate to the data set itself, or serve as a motivation for the inclusion of the kind of data or feature referenced. The large majority of the data used in the EbA priority areas analysis is publically available at the national level in South Africa and could be readily applied to all 44 of South Africa’s rural and peri-urban municipalities. There are only a very small number of place-specific datasets, all of which are used to describe the particular local context and environmental dependencies in the social demand category. For replication outside of South Africa, the four broad categories of information could be applied, but would have to be populated with nationally or locally available data, clipped to the relevant political or administrative boundary for land-use decision-making.

**Table 1:** Summary of natural and socio-economic features included, and data sets which were used to map these, for ecosystem priority areas mapping. Features shown in **bold** were specific to the Namakwa District municipality only; Features shown in *italics* were specific to the Alfred Nzo District municipality only. Further details on all of the mapping methods for each individual layer and for the composite maps of each category can be found in the vulnerability assessment technical reports for each pilot site [44,45].

EbA Map Component	Data Source	Features mapped
**Water Ecological Infrastructure**	A variety of national topocadastral datasets on rivers, wetlands and runoff including from the Surveyor General of South Africa, and the ProEcoServe project (www.proecoserve.org)	The features considered were the ability of a water source to provide (i) water production, (ii) stream flow augmentation, (iii) flood attenuation, (iv) erosion control, (v) water quality [47], (vi) level of transformation [46]
**Important Biodiversity**	South African National Biodiversity Assessment [48]; National Estuary Biodiversity Plan [49]; National Freshwater Ecosystem Priority Areas [50]; Namakwa District Biodiversity Sector Plan [51]; National Protected Areas Expansion Strategy [52,53]	The features considered were (i) aquatic priorities, (ii) critical biodiversity areas, (iii) presence of threatened ecosystems, (iv) ecosystem protection levels, (v) protected area expansion needs, (vi) level of transformation [46]
**Natural features important for climate resilience**	Vegetation of South Africa, Lesotho, and Swaziland [32]; Regions of floristic endemism in South Africa [54]; Layers developed by Holness for National Protected Areas Expansion Strategy [52]	The features considered were (i) coastal corridors [55,56], (ii) riparian corridors and buffers [57,58], (iii) temperature, rainfall and altitudinal gradients [59–62], (iv) areas with high biotic diversity [63–65], (v) centres of floral endemism [66–68], (vi) local species refugia [69–71], (vii) large unfragmented landscapes [65,72,73], (viii) level of transformation [46]
**Socio-economic features of the system**	National Census data collected in 2011 [74]; Location of buildings from Eskom data	The features considered were (i) population densities, (ii) population location, (iii) proportions of low income households, (iv) levels of dependency, (v) access to services, (vi) ownership of goods, (vii) dependency on ground or surface water (not piped), (viii) **dependency on communal rangelands,** (ix) *dependency on wood for fuel*, (x) *dependency on natural building materials*, (xi1) level of transformation [46]

The composite maps produced for water, biodiversity, climate resilience, and socio-economic features were produced in a compatible format for comprehensive coverage at the required scale and scored in a consistent fashion with lowest value areas scoring 0 and highest value areas scoring 10 to allow for later integration into a single EbA priority areas map for each case study site. The authors recognise that quite different variables have been combined, but argue that all are directly relevant for identifying priority areas for EbA implementation. Care was taken to ensure that the data ranges and levels of priority that these ranges represent in the different summary layers were sufficiently similar to allow them to be sensibly combined, with each layer representing a consistent range from lowest priority through to highest priority for EbA implementation.

### The Case Study Sites

**Fig 3:**
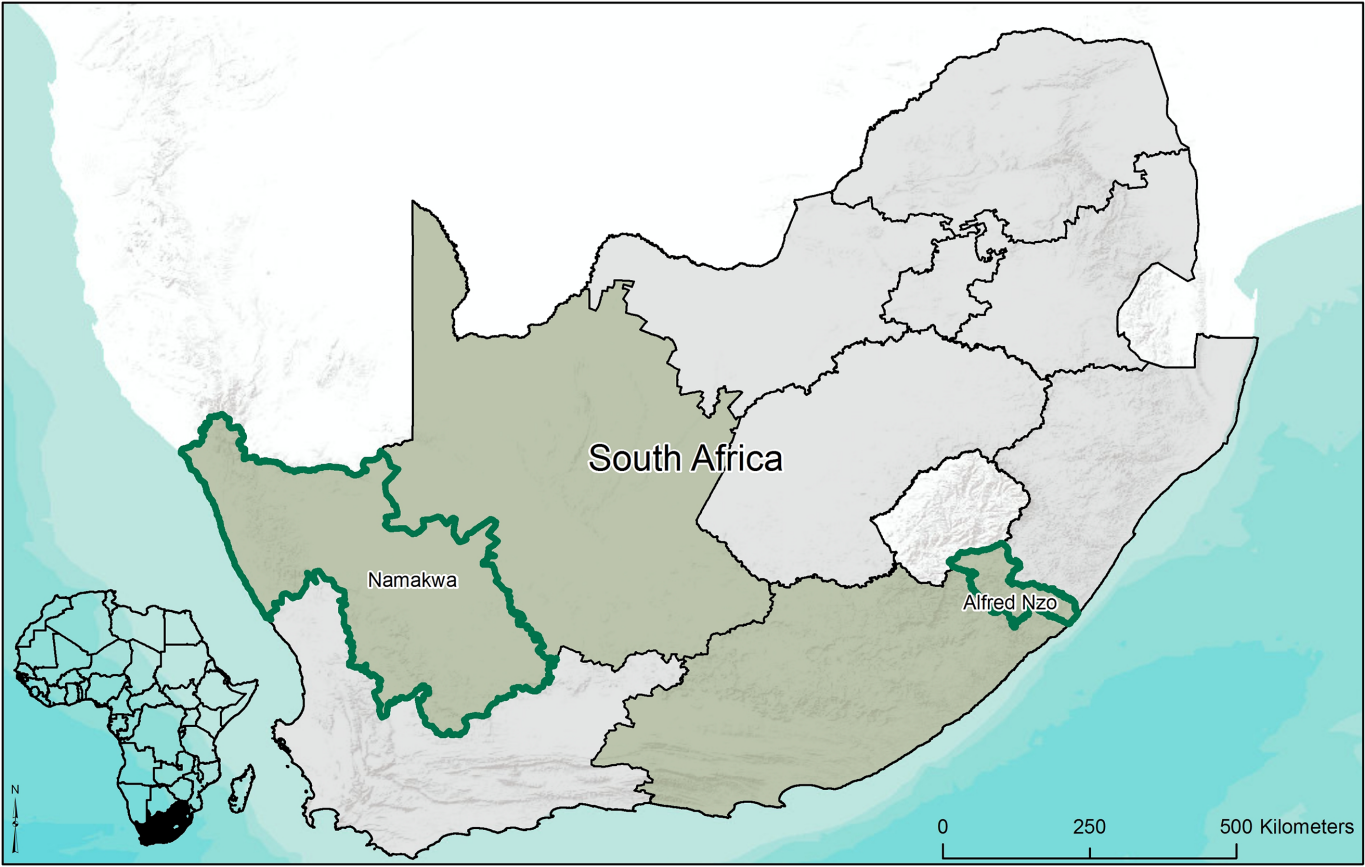
Map showing the location of the two case study sites.

The Namakwa District Municipality (126 860 km2) is located in the northwestern Northern Cape Province in South Africa (Fig 3). It is home to roughly 115,840 people, and much of the Succulent Karoo global biodiversity hotspot [75,76], characterised by succulent shrubland. Climatic conditions are semi-arid, characterised by low mean annual rainfall [77] and extreme temperatures [78].

The Alfred Nzo District Municipality (11,119 km^2^) is located in the northeastern Eastern Cape Province in South Africa. It is located in the Maputoland-Pondoland-Albany global biodiversity hotspot [75,76], characterised by high altitude grasslands and some coastal belt forests, and home to roughly 804,500 people. Climate conditions are sub-humid, with relatively high summer rainfall and temperate conditions [74].

Although spanning a wide range of climatic and socio-economic conditions, both Districts are rural and remote, with limited human and financial resources. Both exhibit limited economic opportunities, extensive dependence on economically marginal agricultural activities and social grants, and high levels of unemployment and poverty [79–81]. Both are, however, critical loci of decision-making authority for service delivery, as well as land use and infrastructure planning. Biome stability and EbA priority areas maps were produced for each case study site as part of formal local government climate change response planning [44,45].

## Results and Discussion

The results of applying the methodological approach to the NDM and the ANDM in South Africa, presented here, serve as an example of how the approach could be applied elsewhere, and demonstrate the products of the analysis that could be expected through replication, i.e. the biome stability and EbA priority areas maps.

### Climate envelope stability at the biome scale

In both the NDM and the ANDM temperatures are projected to increase in the medium term, and substantially so in the longer term (S1 Appendix). Projections for rainfall were divergent, with some models predicting a drier future and others a wetter future, but also well within the current, very wide, range of normal (S1 Appendix). The scenario planning approach used enables decision makers to work with such uncertainty by visualising a range of plausible climate futures that adaptation responses should be able to adequately address. The biome stability maps clearly demonstrate that rising temperatures are an actionable climate change threat, regardless of what happens with rainfall, which is much more difficult to predict, as a result of climate change. This is confirmed in LTAS [43] where all analyses consistently predicted warming in the region alongside uncertainty in terms of rainfall change.

The MaxEnt biome modeling results were extremely consistent for both the medium and longer term across all three climate models, demonstrating that rising temperatures are likely to be a significant driver of vegetation change in both regions. Biome stability maps provide valuable insight into the extent to which ecosystems themselves are vulnerable to climate change and thus the extent to which they can be relied on to provide the suite of ecosystem services on which people currently depend into the future [82]. This is particularly important in rural areas where the economy is linked to agricultural activities, themselves inextricably tied to biomes and their associated vegetation. These projections need to be confirmed through long term monitoring of actual vegetation change under changing climate conditions.

In the NDM, rising temperatures consistent with historical trends [83] are likely, over time, to drive the southward expansion of the Desert Biome into the summer rainfall Nama Karoo biome, currently a commercially productive livestock farming system. The Succulent Karoo biome in the NDM appears stable under all climate change scenarios largely because its current climate envelope is so unique and internally diverse. In the ANDM, rising temperatures and a resultant reduction in the number of frost days are likely to drive a dramatic long-term climate envelope shift from current suitability for highly productive Grassland to a less agriculturally productive Savanna system. This process is likely to exacerbated by the direct effects of rising CO_2_ on tree and bush encroachment [84,85], although this is not simulated by the MaxEnt modelling approach. Visualising these potential shifts in climate suitability for biomes in rural areas primarily engaged in agriculture has been a valuable communication tool, enabling decision-makers to understand and interpret climate risk. The potential for biome climate envelopes to shift in future introduces an element of risk which local decision-makers need to understand in order to plan for and effectively manage change. In Grassland/Savanna systems active management by fire and browsing/grazing offer opportunities to land users to direct vegetation structure and function if the processes are understood.

There are many countries where downscaled climate change information is not available. In those cases, these biome stability maps could only be reproduced using more generic modelling approaches that may offer some insights [86,87]. It nonetheless remains critical to translate the available climate change information into terms that are relevant for local level decision-making in order to place EbA priority areas maps, which do not require downscaled climate impacts information, into context. Narrative descriptions of the likely impacts of climate change on agriculture and other social and economic undertakings in the focus area can be undertaken to provide this context in place of the biome stability mapping exercise.

**Fig 4:**
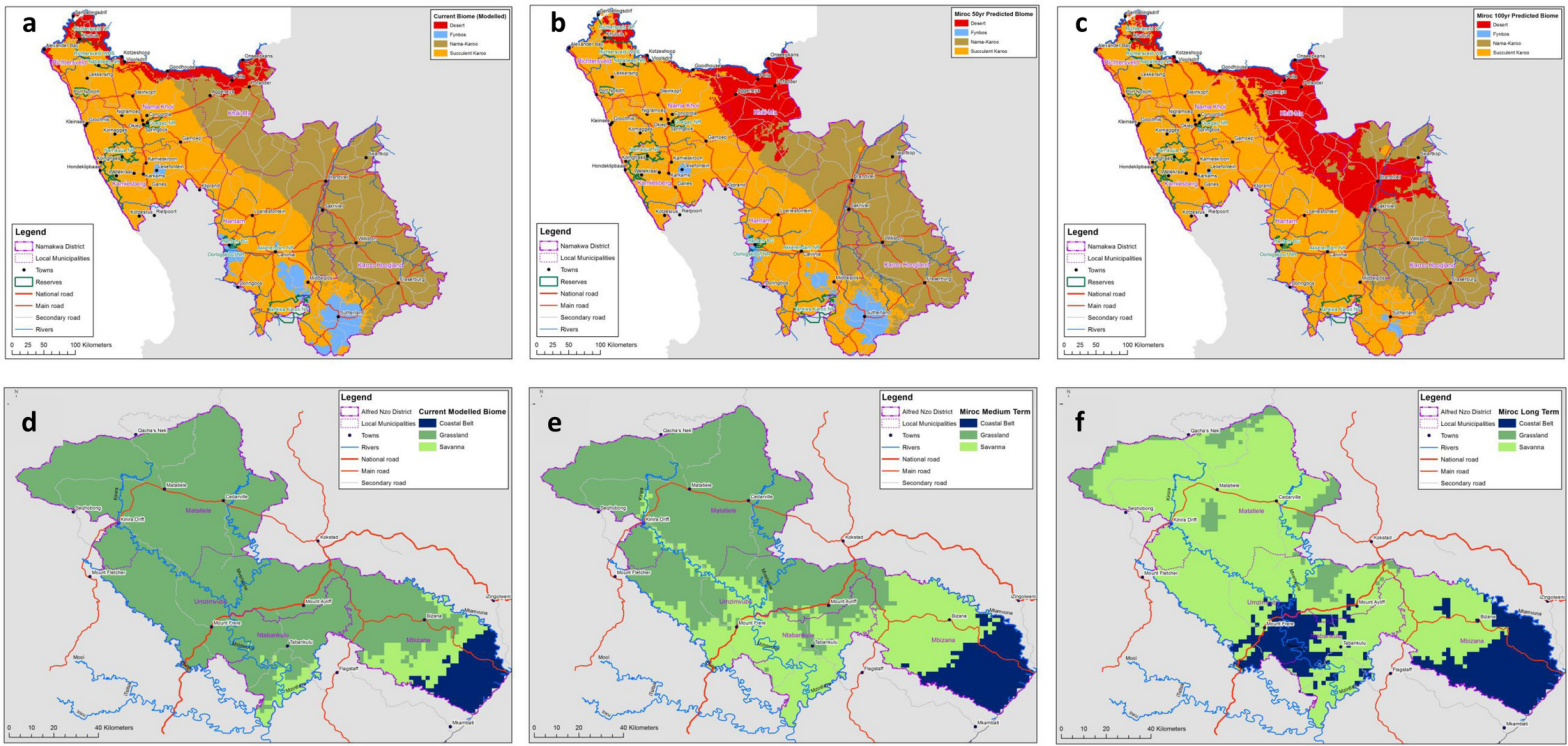
Maps of biome impacts. Modelled current biomes in the NDM (4a), projected changes in climatic suitability for the biomes in the NDM, medium term MIROC (4b), projected changes in climatic suitability for the biomes on the NDM, long term MIROC (4c), modelled current biomes in the ANDM (4d), projected changes in climatic suitability for the biomes in the ANDM, medium term MIROC (4e), projected changes in climatic suitability for the biomes on the ANDM, long term MIROC (4f). Reprinted from [44,45] under a CC BY license, with permission from CSA, original copyright 2015.

**Fig 5:**
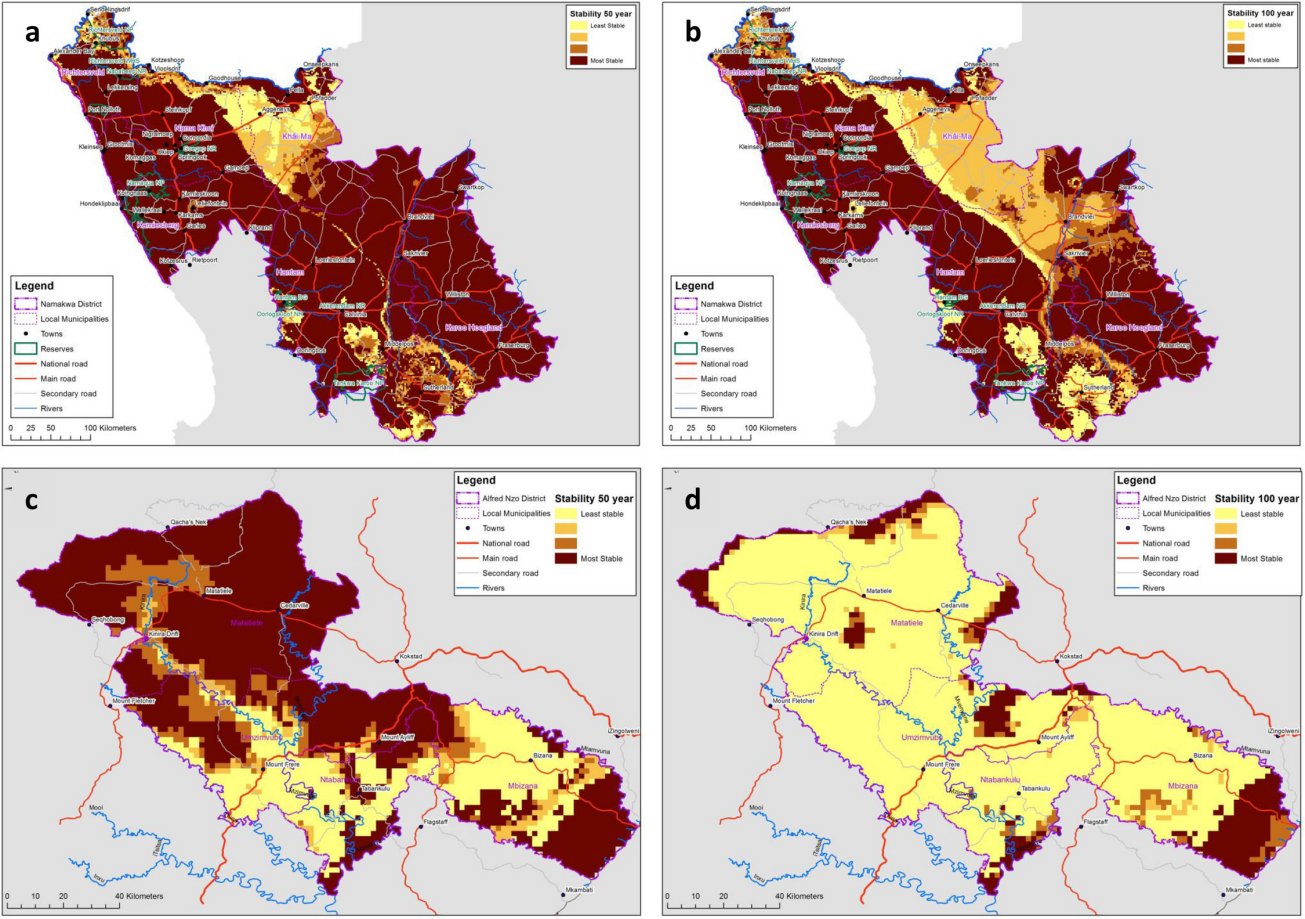
**Map of projected biome climatic envelope stability** over the medium term in the NDM (5a) and longer term in the NDM (5b), as well as over the medium term in the ANDM (5c) and longer term in the ANDM (5d). Reprinted from [44,45] under a CC BY license, with permission from CSA, original copyright 2015.

Fig 4 shows modelled changes in biome distribution, applying MIROC, for both the NDM and the ANDM. Fig 5 shows projected biome climatic envelope stability in the NDM and the ANDM for the longer term, averaged across all models, highlighting the potentially dramatic effect of rising temperatures on future biome stability in some areas.

The biome stability models incorporate some uncertainties that must be clearly communicated. Each biome has a characteristic climate envelope–a range and pattern of temperature and rainfall values–within which it usually occurs. According to our understanding of climate control of vegetation types, as the climate changes, an area that is currently climatically suited to one biome might become more climatically suited to another different biome, inducing climate-related stress in components of the biome. If such changes were to occur over a long period of time (many thousands of years), and if natural habitat were predominantly intact, the ecosystems and species that make up the biome may well be able to undergo adaptation and/or spatial shifts in response [88,89]. However, with changes in climate happening over relatively short periods (decades) and with much natural habitat lost, degraded or fragmented, it is more likely that disruptive change will occur alongside shifts in biome climatic suitability [63,69]. Some biomes, notably the Succulent Karoo in this analysis, encompass a fairly broad range of climatic conditions. Therefore, a specific site could experience fairly large changes in precipitation and temperature while still remaining within the broad envelope of climate conditions currently associated with that biome.

Biome stability modelling provides important broad-scale insights into more and less stable areas under a range of future climate scenarios. As a first step in planning for EbA at the local level, these maps highlight spatially the areas that are likely to remain more stable under a range of possible future climates, and those that are more sensitive to climatic change, in the medium and longer term. In areas where biomes are most at risk of ecological composition and structural change, it is particularly important to retain natural features in the landscape that will allow ecosystems and species to adapt as naturally as possible. Biomes that are more likely to be stable in the face of climate change are more likely to retain their current composition and structure and thus to continue to provide adaptation services to people in the future.

Only three Global Climate Models were used in this analysis and for only one emissions scenario. The climate data used for this analysis were taken from a national dataset and averaged for the case study Districts. The scenarios and models used for the climate analysis are from IPCC AR4 and could be criticized as being outdated. Outputs from LTAS and IPCC AR5 are, however, not significantly different from the models used in this analysis.

A scenario planning and spatial mapping approach for communicating results of a climate change impacts analysis on biome stability was used to highlight climate change impacts as well as areas of remaining uncertainty and to illustrate to local level planners that adaptation actions can be implemented now. Finely downscaled climate data is not essential for identifying EbA priorities and the identification of these priority areas does not include any climate data. It is, rather, important to contextualise these priorities within potential impacts of climate change, to inform and build capacity for implementation.

Each element used to identify priority areas for EbA, designed to speak to local government priorities and mandates around poverty alleviation, is discussed in detail below.

### Identifying water, biodiversity, and climate resilience natural features important for adaptation

In both case studies, the highest value locations for EbA are those sites for water, biodiversity, and climate resilient natural features that follow the escarpment and other important high-lying water catchment areas, the riparian areas, as well as coastal and estuarine areas and unfragmented landscapes linked to large national parks and provincial nature reserves.

### Identifying socio-economic hotspots of local vulnerability to climate change

**Fig 6:**
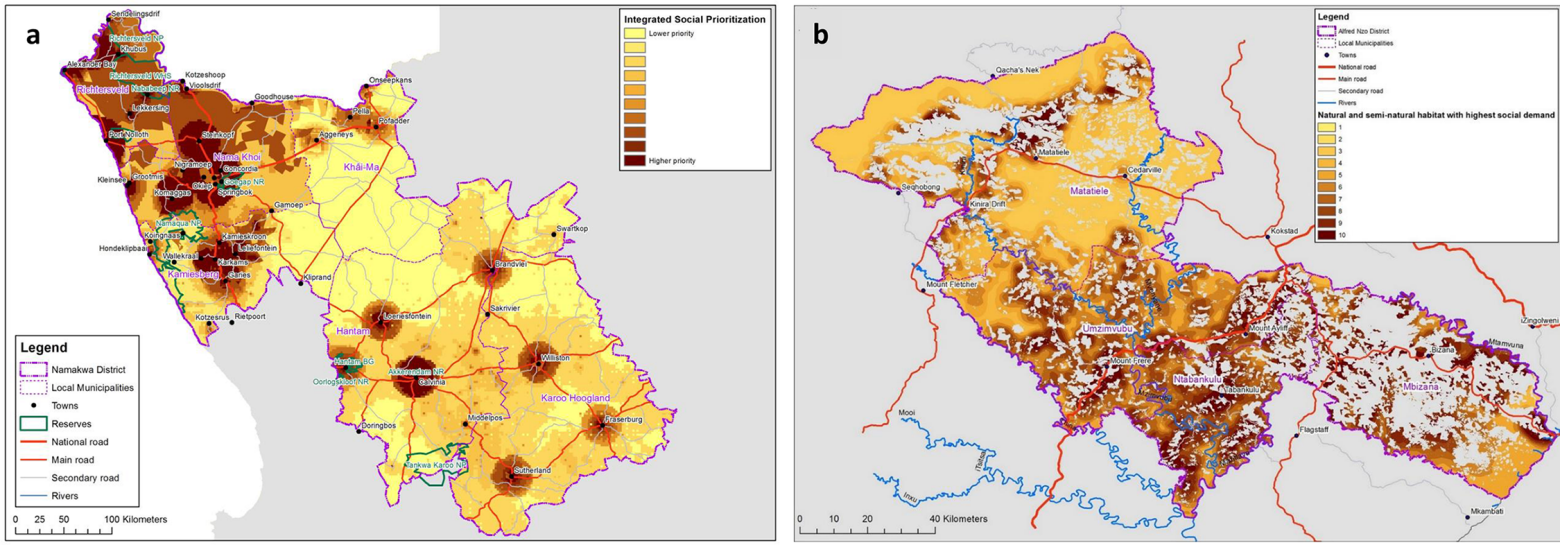
Maps showing areas of high social demand on the environment overlaying Population Density, General Poverty, and Specific Environmental Dependency indicators with natural and semi-natural areas data. The darker areas show those natural and semi-natural areas with high social demand on the environment. These are the areas which need to be most carefully managed to ensure the ongoing supply of good quality environmental goods and adaptation services for people in the NDM (6a) and ANDM (6b). Reprinted from [44,45] under a CC BY license, with permission from CSA, original copyright 2015.

People in the NDM and the ANDM live in scattered and isolated small rural towns and settlements with large areas of sparsely populated farmland between them. The darker areas on the maps shown in Fig 6 indicate lower incomes, lower access to goods and services and higher dependency ratios, i.e. higher levels of poverty, as well as highlighting those natural and semi-natural areas where people are most directly dependent on the environment for their livelihoods.

Most of the data used was sourced directly from the national Census. This allows for direct replication in other South African sites and a generalisable method for spatially identifying nodes of socio-economic vulnerability and environmental dependency. However, it is important to note that the analysis was tailored slightly to the local conditions in the NDM and ANDM, as described in the methods section. In the NDM, critical issues for adaptation are population density, dependence on groundwater, and dependence on communal rangeland resources. Although climate change impacts on groundwater are poorly understood, many of these resources are already under pressure from over-abstraction and Schulze [90] suggests that groundwater recharge in the NDM may be reduced by up to 30% as a result of climate change impacts on hydrological processes. Some 30% of the District is communal rangeland, where people are most directly dependent on functioning ecosystems for their livelihoods [91,92]. In the ANDM, access to freshwater from surface water resources and access to natural building materials are critical adaptation concerns. Such concerns need to be identified at each site of replication to ensure the local relevance of the products.

The merging of information on population density, poverty, and environmental dependency highlights spatially the most important areas for EbA actions that will directly benefit people, from a socio-economic perspective. These tend to be concentrated in the natural and semi-natural areas surrounding the towns and settlements, where people are located.

### Defining priority areas for Ecosystem-based adaptation

Once all of the summary maps of important water ecological infrastructure, biodiversity, and natural features contributing to climate resilience as well as the socio-economic vulnerability and environmental dependency nodes are produced, these can be equally weighted and combined into a single composite map within which all irreversibly modified landscapes are removed. Through this process, priority areas for the implementation of EbA emerge clearly. The means for identifying sites for EbA implementation lies in the overlap between the social and natural features described in each individual layer of information.

**Fig 7:**
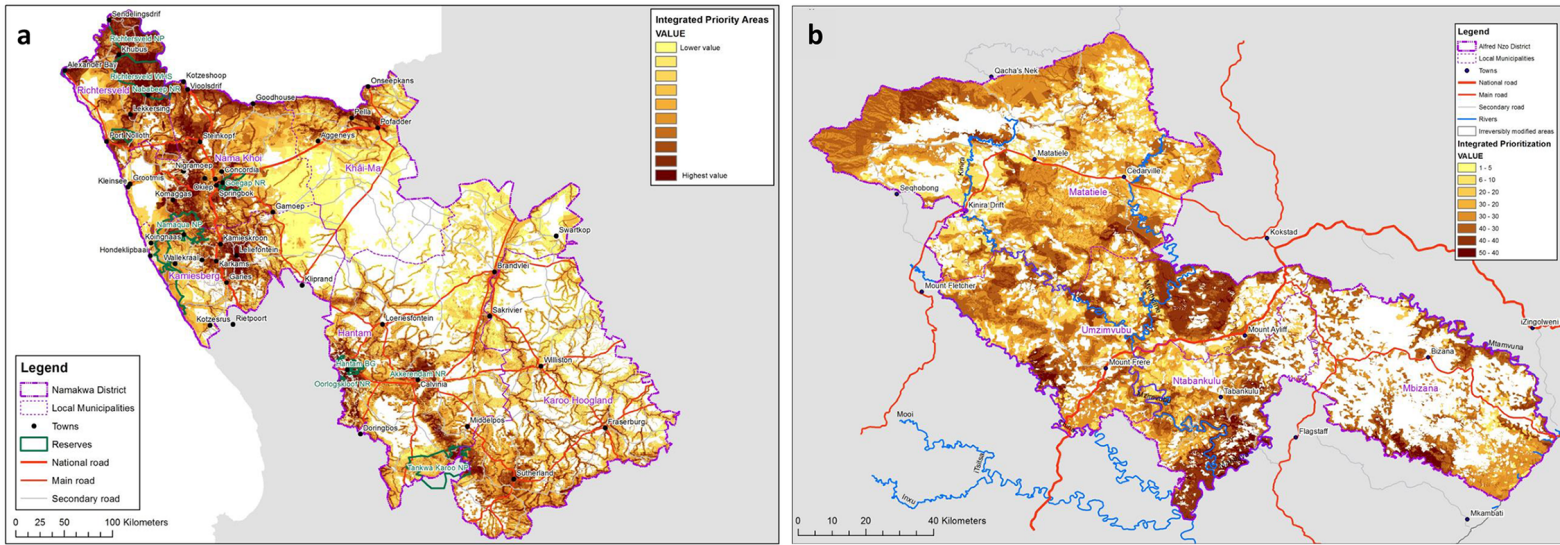
**Integrated map of priority areas for Ecosystem-based Adaptation to climate change impacts in the NDM (7a) and the ANDM (7b).** Reprinted from [44,45] under a CC BY license, with permission from CSA, original copyright 2015.

The EbA priority areas maps, shown in Fig 7, highlight locations in the landscape that are critical for delivering ecosystem services, supporting the overall socio-ecological system. These systems will provide climate change adaptation services in the future and are most important for supporting people who are directly dependent on the natural environment for their livelihoods. These areas scientifically prioritise foci for the restoration, conservation, and sustainable management of ecosystems to ensure ecosystem function as a foundation for climate resilience and the development of the regional green economy, highlighting for decision-makers the places that will derive maximum climate resilience benefit from investments in ecosystems.

In combination, these can be used to prioritise natural and semi-natural areas that are critical for supporting human resilience to climate change in the landscape. Application of this methodology will allow for comprehensive spatial planning and identification of priority areas for implementing EbA actions at the local level, in support of an EbA strategy that is ‘based on maintaining sufficient intact natural habitat in an optimal configuration identified through systematic biodiversity planning’ [93] and incorporating social concerns. The approach also allows for the comprehensive identification of sensitive or important areas for inclusion into a range of other sub-national planning processes.

Local authorities need information at the scale of the political units that they manage. There is limited ability or willingness to take up information when it does not align well with their respective political unit. This includes where the available information does not cover their full area of responsibility and hence needs to be integrated with other data sources to provide a complete picture or where the information extends beyond their jurisdiction and where only a portion is relevant for the decision-maker. An example of this is information presented at a catchment scale. Presenting information at the scale of the sub-national administrative unit enables direct embedding of the priority areas identified into wider government and institutional processes while also allowing for the consideration of both long and short term needs. As the entities most directly responsible for local-level planning and management, local government represent a crucial site for implementing EbA activities [6,10,94–96].

The products presented in this paper, namely biome stability and EbA priority areas maps, provide a spatial mechanism for interpreting, translating, and condensing complex climate, ecological, and socio-economic information at the practical scale of the familiar administrative boundaries within which local government officials operate and have a legal mandate. The products were developed in close collaboration with both Districts. Furthermore, the products have been actively taken up for developing local level climate strategies and related climate change adaptation planning. In the NDM, the EbA priority areas map has been used to identify several natural resources management project implementation sites and is included in the 2014 District Integrated Development Plan (IDP) [97], the guiding document for local government activity. In the ANDM, these products are included in the IDP and formed the basis of a District Climate Change Response Strategy [98]. Details on the stakeholder engagement process and level of uptake is the subject of a forthcoming paper.

The methodological approach for identifying EbA priority areas, overlaying environmental and demographic data using standard multivariate GIS analyses and publically available national data sets, can be quickly adjusted to be applicable throughout South Africa, for any of the 44 non-metropolitan Districts. The overall approach could usefully be applied at the same sub-national scale for local authorities throughout much of the developing world, where the necessary basic biodiversity and socio economic information exist. The appropriate level of government will vary in other countries and information should be collated at the locally appropriate scale for adaptation related decision-making. Our analysis is particularly well-suited for rural and peri-urban areas, where large numbers of people are directly dependent on natural resources for their livelihoods.

The current assessment represents a major step forward in the representation of climate change vulnerability and resilience spatially, in an accessible, yet still comprehensive format. As a next step, spatially defined priority areas need to be linked explicitly with clear, site-specific implementation activities. This is best achieved through a participatory planning process. CARE [99], DEA [100], and Oettle et al [101] have produced useful examples of facilitated processes at various scales relevant for operationalising the information presented here. Participatory and stakeholder engaged local work is needed to identify what EbA actions to implement, when these are the most appropriate actions, and to what extent they can be effective, as well the actual limits of ecosystems in terms of the extent of climatic change they themselves can withstand.

## Conclusion

The two spatial tools presented here have been developed specifically to support local government decision-makers with the allocation of scarce resources for climate change response in localities that have the potential to deliver the maximum return on investment in terms of biodiversity conservation, ecosystem services provision, and contribution to local livelihoods. Some features in the landscape are likely to contribute more to climate resilience than others. Concentrating restoration, protection and land management activities in these EbA priority areas will likely maximise the potential for natural ecosystems to contribute to building social and ecological resilience to climate change, ultimately reducing the vulnerability of the people living in these highlighted areas.

The EbA priority areas maps define sites for the restoration and conservation of scientifically prioritised landscapes to ensure ecosystem function as a foundation for climate resilience and the development of the regional green economy. The methodology provides localised site specific analysis, guidance and recommendations to local decision makers and managers regarding the possible impacts of climate change in the district as well as the spatial priority areas in which EbA efforts could be concentrated. Mapping the spatial distribution of social resources, such as access to basic services or ownership of assets, as well as ecological resources, such as areas critical for supplying ecosystem services and supporting climate resilience, provides an opportunity for decision makers at the local level to plan and implement win-win adaptation interventions.

Using industry standard software, publically available data, and straightforward methodological approaches allows for easy updating, revision, refinement, and replication. The approach can be replicated widely, in South Africa and other countries, with minor adjustments to accommodate for site specific variations in socio-ecological systems. With a growing recognition globally that intact biodiversity is an important requirement for human adaptation to climate change [93], particularly for developing countries, these tools are useful for identifying the areas that have the highest potential to help local communities adapt to climate change by protecting, restoring and managing the ecosystems on which local livelihoods depend.

## Supporting Information

S1 Appendix Current and modelled seasonal and annual temperature and precipitation for the NDM and the ANDM. (DOCX)

## References

[1] IPCC (Intergovernmental Panel on Climate Change). Climate Change 2014: Impacts, Adaptation, and Vulnerability Part A: Global and Sectoral Aspects. Contribution of Working Group II to the Fifth Assessment Report of the Intergovernmental Panel on Climate Change Field CB, Barros VR, Dokken DJ, Mach KJ, Mastrandrea MD, Bilir TE et al (eds). Cambridge, Cambridge University Press; 2014.

[2] IPCC (Intergovernmental Panel on Climate Change. Climate Change 2014: Impacts, Adaptation, and Vulnerability Part B: Regional Aspects. Contribution of Working Group II to the Fifth Assessment Report of the Intergovernmental Panel on Climate Change Barros VR, Field CB, Dokken DJ, Mastrandrea MD, Mach KJ, Bilir TE, et al (eds). Cambridge, Cambridge University Press; 2014.

[3] Jones HP, Hole DG. Harnessing nature to help people adapt to climate change. Nat Clim Chang 2012; 2: 504–509.

[4] Munroe R, Roe D, Doswald N, Spencer T, Möller I, Vira B, et al Review of the evidence base for ecosystem-based approaches for adaptation to climate change. Environ Evid. 2012; 1: 13.

[5] Secretariat of the Convention on Biological Diversity. Connecting Biodiversity and Climate Change Mitigation and Adaptation: Report of the Second Ad Hoc Technical Expert Group on Biodiversity and Climate Change. Montreal; 2009; Technical Series No. 41.

[6] Pasquini L, Cowling R. Opportunities and challenges for mainstreaming ecosystem-based adaptation in local government: evidence from the Western Cape South Africa. Environ Dev Sust 2015; 17(5):1121–1140.

[7] Leichenko RM, O’Brien KL. The dynamics of rural vulnerability to global change: the case of Southern Africa. Mitig Adapt Strat Gl. 2002; 7: 1–18.

[8] Ludi E, Wiggins S, Jones L, Lofthouse J, Levine S. Adapting development: How wider development interventions can support adaptive capacity at the community level In Schipper ELF, Ayers J,. Reid H, Huq S, Rahman A. editors. Community-based Adaptation to Climate Change: Scaling it up. New York, Routledge; 2014.

[9] Walker B, Holling CS, Carpenter S, Kinzig A. Resilience, adaptability, and transformability in social-ecological systems. Ecol Soc 2004; 9(2):5.

[10] Pasquini L, Cowling RM, Ziervogel G. Facing the heat: Barriers to mainstreaming climate change adaptation in local government in the Western Cape Province, South Africa. Habitat Int 2013; 40: 225–232.

[11] Reid H. Ecosystem-and community-based adaptation: learning from community-based natural resource management. Clim Dev. 2015; 10.1080/17565529.2015.1034233

[12] Travers A, Elrick C, Kay R, Vestergaard O. Ecosystem-based Adaptation Guidance: Moving from principles to practice. UNEP working document 2012. Available: http://www.unep.org/climatechange/adaptation/Portals/133/documents/Ecosystem-Based%20Adaptation/Decision%20Support%20Framework/EBA%20Guidance_WORKING%20DOCUMENT%2030032012.pdf.

[13] Doswald N, Estrella M. Promoting ecosystems for disaster risk reduction and climate change adaptation: opportunities for integration United Nations Ecosystem Programme 2015 Available: http://www.unep.org/disastersandconflicts/portals/155/publications/EcoDRR_Discussion_paper_web.pdf.

[14] Rannow S, Loibl W, Grieving S, Gruehn D, Meyer B. Potential impacts of climate change in Germany–identifying regional priorities for adaptation activities in adaptation planning. Landsc Urban Plan 2010; 98: 160–171.

[15] De Sherbinin A. Climate change hotspots mapping: what have we learned? Clim Change. 2014; 123: 23–37.

[16] Doswald N, Munroe R, Roe D, Giuliani A, Castelli I, Stephens J, et al Effectiveness of ecosystem-based approaches for adaptation: review of the evidence-base. Clim Dev. 2014; 6(2): 185–201.

[17] Egoh B, Reyers B, Rouget M, Richardson DM, Le Maitre DC, van Jaarsveld AS. Mapping ecosystem services for planning and management. Agric Ecosyst Environ. 2008; 127: 135–140.

[18] Metzger MJ, Schröter D, Leemans R, Cramer W. A spatial explicit and quantitative vulnerability assessment of ecosystem services change in Europe. Reg Environ Change 2008; 8: 91–107.

[19] Burkhard B, Kroll F, Nedkov S, Müller F. Mapping ecosystem service supply, demand and budgets. Ecol Indic 2012; 21: 17–29.

[20] Alessa L, Kliskey A, Brown G. Social–ecological hotspots mapping: a spatial approach for identifying coupled social–ecological space. Landsc Urban Plan. 2008; 85, 27–39.

[21] Hamann M, Biggs R, Reyers B. Mapping social–ecological systems: Identifying ‘green-loop’ and ‘red- loop’ dynamics based on characteristic bundles of ecosystem service use. Glob Environ Change 2015; 34: 218–226.

[22] Cumming GS, Buerkert A, Hoffmann EM, Schlecht E, von Cramon-Taubadel S., Tscharntke T. Implications of agricultural transitions and urbanization for ecosystem services. Nature 2014; 515: 50–57. 10.1038/nature13945 25373674

[23] Margules CR, Pressey RL. Systematic conservation planning. Nature 2000; 405: 243–253. 10821285 10.1038/35012251

[24] Pramova E, Locatelli B, Djoudi H, Olufunso AS. Forests and trees for social adaptation to climate variability and change. WIREs Clim Change 2012; 3(6): 581–596.

[25] Roberts D, Boon R, Diederichs N, Douwes E, Govender N, Mcinnes A, et al Exploring ecosystem-based adaptation in Durban, South Africa: “learning-by-doing” at the local government coal face. Environ Urban. 2012; 24: 167–195.

[26] Ruckelshaus M, Doney SC, Galindo HM, Barry JP, Chan F, Duffy JE, et al Securing ocean benefits for society in the face of climate change. Mar Policy 2013; 40: 154–159.

[27] Magadlela D, Mdzeke N. Social benefits in the Working for Water programme as a public works initiative: working for water. S Afr J Sci. 2004; 100, 94–96.

[28] Carrick PJ, Erickson TE, Becker CH, Mayence CE, Bourne AR. Comparing ecological restoration in South Africa and Western Australia: the benefits of a travelling workshop. Ecol Manage Restor 2015; 16(2) 10.1111/emr.12169

[29] Elith J, Phillips S, Hastie T, Dudik M, Chee Y, Yates CJ. A statistical explanation of MaxEnt for ecologists. Divers Distrib. 2010; 17(1): 43–57.

[30] Warren DL, Seifert SN. Ecological niche modeling in Maxent: the importance of model complexity and the performance of model selection criteria. Ecol Appl 2011; 21:335–342. 21563566 10.1890/10-1171.1

[31] Fourcade Y, Engler JO, Rodder D, Secondi J. Mapping species distributions with MAXENT using a geographically biased sample of presence data: A performance assessment of methods for correcting sampling bias. PLoS One 2014; 9(5). doi: 10.1371 10.1371/journal.pone.0097122 PMC4018261 24818607

[32] Mucina L, Rutherford MC. The Vegetation of South Africa, Lesotho and Swaziland. Pretoria: South African National Biodiversity Institute, Pretoria, South Africa 2006.

[33] Araújo MB, Peterson AT. Uses and misuses of bioclimatic envelope modelling. Ecology. 2012; 93: 10.1890/11-1930.1 22919900

[34] Engelbrecht FA. Simulations of climate and climate change over Southern and tropical Africa with the conformal-cubic atmospheric model In Schulze RE (Ed) Climate Change and Water Resources in Southern Africa: Studies on Scenarios, Impacts, Vulnerabilities and Adaptation. Pretoria: Water Research Commission 2005 pp57–74.

[35] Schulze RE, Lumsden TG, Horan MJC, Warburton M, Maharaj M. An assessment of impacts of climate change on agrohydrological responses over southern Africa. In Schulze RE (Ed) Climate Change and Water Resources in Southern Africa: Studies on Scenarios, Impacts, Vulnerabilities and Adaptation. Water Research Commission: Pretoria, RSA WRC Report 1430/1/05. Chapter 9; 141–189; 2005.

[36] Schulze RE. Atlas of Climate Change and the South Africa Agricultural Sector: A 2010 Perspective. Pretoria: Department of Agriculture, Forestry and Fisheries; 2010.

[37] Nozawa T, Nagashima T, Ogura T, Yokohata T, Okada N, Shiogama H. Climate change simulations with a coupled ocean-atmosphere GCM called the Model for Interdisciplinary Research on Climate: MIROC CGER Supercomputer Monograph Report Vol. 12 Japan: National Institute for Environmental Studies; 2007.

[38] Roeckner E, Bauml G, Bonaventura L, Brokopf R, Esch M, Giorgetta M, et al The atmospheric general circulation model ECHAM5: Model description Germany: Max Planck Institute for Meteorology Report No. 349; 2003.

[39] Stevens B, Giorgetta M, Esch M, Mauritson T, Crueger T, Rast S, et al Atmospheric component of the MPI-M Earth System Model: ECHAM6. J Adv Model Earth Sy. 2013; 5(2): 146–172.

[40] Gordon H, O’Farrell S, Collier M, Dix M, Rotstayn L, Kowlaczyk E, et al. The CSIRO Mk 3.5 Climate Model. The Centre for Australian Weather and Climate Research Technical Report No. 21. Australia. ISSN: 1836-019X. 2010.

[41] Engelbrecht FA, McGregor JL, Engelbrecht CJ. Dynamics of the Conformal-Cubic Atmospheric Model projected climate change signal over southern Africa. Int J Climatol. 2008; 29: 1013–1033

[42] IPCC (Intergovernmental Panel on Climate Change). Emissions Scenarios: Summary for policymakers. IPCC Special Report of Working Group 3. ISBN: 92-9169-113-5. 2000.

[43] DEA (Department of Environmental Affairs). Long-Term Adaptation Scenarios Flagship Research Programme (LTAS) for South Africa. Climate Trends and Scenarios for South Africa. Pretoria, South Africa. 2013

[44] Bourne A, de Abreu P, Donatti C, Holness S, Scorgie S, Midgley GF. Climate Change: Vulnerability Assessment for the Namakwa District Municipality. Conservation South Africa. 2015 Available: http://www.conservation.org/publications/Documents/CI-CASCADE-Namakwa-Vulnerability-Assessment.pdf.

[45] Conservation South Africa. Climate Change Vulnerability Assessment: Alfred Nzo Municipality. 2015. Available: https://www.dropbox.com/s/1minhkiskysv5ly/27%20March%202015%20Final%20Draft.zip?dl=0.

[46] SANBI (South African National Biodiversity Institute). Updating National Landcover. 2009. Available: http://bgis.sanbi.org/landcover/Landcover2009.pdf.

[47] Kotze D, Marnewick G, Batchelor A, Lindley D, Collins N. WET-EcoServices. Water Research Commission Report Number TT 339/09; 2009.

[48] Nel JL, Driver A, Swartz ER. National Biodiversity Assessment 2011: Technical Report. Volume 2: Freshwater Component. CSIR Report Number CSIR/NRE/ECO/IR/2012/0022/A. Stellenbosch: Council for Scientific and Industrial Research; 2012.

[49] Turpie JK, Wilson G Van Niekerk L. National Biodiversity Assessment 2011: National Estuary Biodiversity Plan for South Africa Anchor Environmental Consulting, Cape Town. Report produced for the Council for Scientific and Industrial Research and the South African National Biodiversity Institute 2012.

[50] Nel JL, Driver A, Strydom WF, Maherry A, Petersen C, Hill L, et al. Atlas of Freshwater Ecosystem Priority Areas in South Africa. WRC Report No. TT 500/11. Pretoria: Water Research Commission; 2011.

[51] Northern Cape Province. Namakwa Biodiversity Sector Plan. 2008. Available: http://bgis.sanbi.org/namakwa/project.asp.

[52] Government of South Africa. National Protected Areas Expansion Strategy for South Africa 2008. Pretoria, South Africa.2008. Available: http://bgis.sanbi.org/protectedareas/Nationa_Protected_Area_Expansion_Strategy.pdf.

[53] Holness SD, Biggs HC. Systematic conservation planning and adaptive management. Koedoe 2011; 53(2) 10.4102/koedoe.v53i2.1029

[54] Van Wyk A, Smith G. Regions of floristic endemism in Southern Africa Hatfield: Umdaus Press; 2001.

[55] Heller NE, Zavaleta ES. Biodiversity management in the face of climate change: a review of 22 years of recommendations. Biol Conserv. 2009; 142(1): 14–32.

[56] Klein RJT, Nicholls RJ, Ragoonaden S, Capobianco M, Aston J, Buckley EN. Technological options for adaptation to climate change in coastal zones. J Coast Res 2001; 17(3): 531–543.

[57] Erwin KL. Wetlands and global climate change: the role of wetland restoration in a changing world. Wet Ecol Manag. 2009; 17: 71–84.

[58] Seavy NE, Gardali T, Golet GH, Griggs FT, Howell CA, Kelsey R, et al Why climate change makes riparian restoration more important than ever: recommendations for practice and research. Ecol Restor. 2015; 27(3): 330–338.

[59] Colwell RK, Brehm G, Cardelus CL, Gilman AC, Longino JT. Global warming, elevational range shifts, and lowland biotic attrition in the wet tropics. Science 2008; 322(5899): 258–261. 10.1126/science.1162547 18845754

[60] Walther G-R, Post E, Convey P, Menzel A, Parmesan C, Beebee TJC., et al Ecological responses to recent climate change. Nature 2002; 416: 389–395. 11919621 10.1038/416389a

[61] Wilson RJ, Gutierrez D, Gutierrez J, Martinez D, Agudo R, Monserrat V. Changes to the elevation limits and extent of species ranges associated with climate change. Ecol Lett 2005; 8(11): 1138–1146. 10.1111/j.1461-0248.2005.00824.x 21352437

[62] Sgro CM, Lowe AJ, Hoffmann AA. Building evolutionary resilience for conserving biodiversity under climate change. Evol Appl 2011; 4(2): 326–337. 25567976 10.1111/j.1752-4571.2010.00157.x PMC3352557

[63] Hannah L, Midgley GF, Lovejoy T, Bond WJ, Bush M, Lovett JC, et al Conservation of biodiversity in a changing climate. Conserv Biol. 2002; 16(1):264–268. 10.1046/j.1523-1739.2002.00465.x 35701969

[64] Godfree R, Lepschi B, Reside A, Bolger T, Robertson B, Marshall D, et al (2011). Multiscale topoedaphic heterogeneity increases resilience and resistance of a dominant grassland species to extreme drought and climate change. Glob Chang Biol. 2011; 17(2): 943–958.

[65] Hodgson JA, Thomas CD, Wintle BA, Moilanen A. Climate change, connectivity, and conservation decision-making: back to basics. J Appl Ecol. 2009; 46(5): 964–969.

[66] Cowling RM, Pressey RL, Lombard AT, Desmet PG, Ellis AG. From representation to persistence: requirements for a sustainable system of conservation areas in the species-rich Mediterranean-climate desert of southern Africa. Divers Distrib. 1999; 5(1–2): 51–71.

[67] Klausmeyer KR, Shaw MR. Climate change, habitat loss, protected areas, and the climate adaptation potential of species in Mediterranean ecosystems worldwide. PLoS One 2009; doi: 10.1371 10.1371/journal.pone.0006392 PMC2712077 19641600

[68] Loarie SR, Carter BE, Hayhoe K, McMahon S, Moe R, Knight CA, et al (2008). Climate change and the future of California’s endemic flora. PLoS One 2008; 3(6): e2502 10.1371/journal.pone.0002502 18648541 PMC2481286

[69] Thomas CD, Cameron A, Green RE, Bakkenes M, Beaumont LJ, Collingham YC, et al (2004). Extinction risk from climate change. Nature 2004; 427: 145–148. 14712274 10.1038/nature02121

[70] Ohlemuller R, Anderson BJ, Araujo MB, Butchart SHM, Kudrna O, Ridgley RS, et al The coincidence of climatic and species rarity: high risk to small-range species from climate change. Biol Lett. 2008; 4(5): doi: 10.1098 10.1098/rsbl.2008.0097 PMC2610064 18664421

[71] Dirnbock T, Essl F, Rabitsch W. Disproportional risk for habitat loss of high-altitude endemic species under climate change. Glob Chang Biol. 2011; 17(2): 990–996.

[72] Opdam P, Wascher D. Climate change meets habitat fragmentation: linking landscape and biogeographical scale levels in research and conservation. Biol Conserv. 2004; 117(3):285–296.

[73] Dawson TP, Jackson ST, House JI, Prentice IC, Mace GM. Beyond Predictions: Biodiversity conservation in a changing climate. Science 2011; 332 (6025):53–58. 10.1126/science.1200303 21454781

[74] StatsSA (Statistics South Africa). Census 2011 Statistical Release. Pretoria. 2012. Available: http://www.statssa.gov.za/publications/P03014/P030142011.pdf on 15/5/2015.

[75] Myers N, Mittermeier RA, Mittermeier CG, da Fonseca GAB, Kent J. Biodiversity hotspots for conservation priorities. Nature 2000; 403, 853–858. 10706275 10.1038/35002501

[76] Sloan S, Jenkins CN, Joppa LN, Gaveau DLA, Laurance WF. Remaining natural vegetation in the global biodiversity hotspots. Biol Conserv. 2014; 177: 12–24.

[77] Hoffman M, Carrick P, Gillson L, West A. Drought, climate change and vegetation response in the succulent karoo, South Africa. S Afr J Sci. 2009; 105(1–2): 54–60.

[78] Kelso C, Vogel C. (2007). The climate of Namaqualand in the nineteenth century. Clim Change 2007; 83(3): 357–380.

[79] Cousins B, Hoffman MT, Allsopp N, Rohde RF. A synthesis of sociological and biological perspectives on sustainable land use in Namaqualand. J Arid Environ. 2007; 70: 834–846.

[80] Vetter S. Drought, change and resilience in South Africa's arid and semi-arid rangelands. S Afr J Sci. 2009; 105(1–2):29–33.

[81] DEDEAT (Department of Economic Development, Environmental Affairs and Tourism). The Eastern Cape Socio-economic Review and Outlook. 2013. Available: http://www.dedea.gov.za/research/Research/Socio-Economic%20Review%20and%20Outlook%202013-1.pdf.

[82] Lavorel S, Colloff MJ, Mcintyre S, Doherty MD, Murphy HT, Metcalfe DJ, et al Ecological mechanisms underpinning climate adaptation services. Glob Chang Biol. 2014; 21(1): 12–31. 10.1111/gcb.12689 25131443

[83] Davis C, Hoffman T, Roberts W. Recent trends in the local climate of Namaqualand: a mega-diverse arid region of South Africa. S Afr J Sci. 2016; in press.

[84] Wigley BJ, Bond WJ, Hoffman MT. Bush encroachment under three contrasting land-use practices in a mesic South African savanna. Afr. J. Ecol. 2009; 47:62–70.

[85] Wigley BJ, Bond WJ, Hoffman MT. Thicket expansion in a South African savanna under divergent land use: local vs. global drivers. Glob. Change Biol. 2010; 16(3):964–976.

[86] Moncrieff GR, Scheiter S, Bond WJ, Higgins SI. Increasing atmospheric CO2 overrides the historical legacy of multiple stable biome states in Africa. New Phytol. 2014; 201:908–915. 10.1111/nph.12551 24400901

[87] Moncrieff GR, Scheiter S, Slingsby JA, Higgins SI. Understanding global change impacts on South African biomes using Dynamic Vegetation Models. S Afr. J. Bot. 2015; 10.1016/j.sajb.2015.02.004.

[88] Thuiller W, Albert C, Araujo MB, Berry PM, Cabeza M, Guisan A, et al Predicting global change impacts on plant species’ distributions: future challenges. Perspect Plant Ecol Evol Syst. 2008; 9:137–152.

[89] Thuiller W, Lavergne S, Roquet C, Boulangeat I, Araujo MB. Consequences of climate change on the tree of life in Europe. Nature 2011; 470:531–534. 10.1038/nature09705 21326204

[90] Schulze RE. A 2011 perspective on climate change and the South African water sector. Water Research Commission Report Number 1843/2/11; 2011.

[91] Hoffman MT, Cousins C, Meyer T, Petersen A, Hendriks H. Historical and contemporary agricultural land use and the dersertification of the Karoo In Dean WRJ, Milton S (eds) The Karoo: ecological patterns and processes. Cambridge: Cambridge University Press; 1999.

[92] Samuels MI. Pastoral mobility in a variable and spatially constrained South African environment. PhD thesis, University of Cape Town. 2013.

[93] Petersen C, Holness SD. South Africa: Ecosystem-Based Planning for Climate Change. World Resources Report. 2008. Available: http://www.worldresourcesreport.org.

[94] Huq S, Kovats S, Reid H, Satterthwaite D. Editorial: Reducing risks to cities from disasters and climate change. Environment and Urbanization 2007; 19(1): 3–15.

[95] Measham TG, Preston BL, Smith TF, Brooke C, Gorddard R, Withycombe G, et al Adapting to climate change through local municipal planning: Barriers and challenges. Mitig Adapt Strat Gl 2011; 16(8): 889–909.

[96] Wamsler C, Leuderitz C, Brink E. Local levers for change: Mainstreaming ecosystem-based adaptation into municipal planning to foster sustainability transitions. Glob Environ Change. 2014; 29: 189–201.

[97] Namakwa District Municipality Integrated Development Plan 2013/2014. 2014. Available www.namakwa-dm.gov.za

[98] Conservation South Africa. Alfred Nzo District Municipality Climate Change Response Strategy and Technical Report. 2015. Available here.

[99] CARE International. Climate Vulnerability and Capacity Analysis Handbook. 2009. Available: http://careclimatechange.org/wp-content/uploads/2014/12/CVCA_EN.pdf.

[100] Department of Environmental Affairs, Department of Cooperative Governance, the South African Local Government Association. Let’s Respond: Integrating climate change risks and opportunities into municipal planning. South Africa. 2012. Available https://www.google.co.za/webhp?sourceid=chrome-instant&ion=1&espv=2&ie=UTF-8#q=lets%20respond%20toolkit.

[101] Oettle N, Koelle B, Law S, Parring S, Schmiedel U, Archer van Garderen E, et al Participatory Adaptation Handbook: A practitioner’s guide for facilitating people centred adaptation to climate change. Nieuwoudtville, Indigo Development and Change; 2014.

