# Supplementary material for: A Socio-Ecological Approach for Identifying and Contextualising Spatial Ecosystem-Based Adaptation Priorities at the Sub-National Level
Source: PLoS One. 2016 May 26;11(5):e0155235. doi: 10.1371/journal.pone.0155235 (PMC4881973; doi:10.1371/journal.pone.0155235)
Supplement: S1 Appendix — (DOCX) [file pone.0155235.s001.docx]

S1 Appendix: Current and modelled seasonal and annual temperature and precipitation for the NDM and ANDM

Table: NDM and its local municipalities for the medium term 2040-2059 time period

|  | Area | Spring Rain (mm) | Spring Temperature (°C) | Summer Rain (mm) | Summer Temperature (°C) | Autumn Rain (mm) | Autumn Temperature (°C) | Winter Rain (mm) | Winter Temperature (°C) | Annual Rain (mm) | Annual Temperature (°C) |
| --- | --- | --- | --- | --- | --- | --- | --- | --- | --- | --- | --- |
| Current Climate | Richtersveld | 3.8 | 19.7 | 0.1 | 24.7 | 10.8 | 20.3 | 19.7 | 13.8 | 34.4 | 19.6 |
|  | Nama Khoi | 9.2 | 18.9 | 1.4 | 23.9 | 21.0 | 19.8 | 38.2 | 13.2 | 69.8 | 19.0 |
|  | Kamiesberg | 17.3 | 17.6 | 1.1 | 22.3 | 27.5 | 19.1 | 58.8 | 13.2 | 104.8 | 18.0 |
|  | Hantam | 15.2 | 17.6 | 9.2 | 23.4 | 37.7 | 18.0 | 39.9 | 11.1 | 101.9 | 17.5 |
|  | Karoo Hoogland | 15.8 | 15.6 | 17.8 | 21.9 | 43.5 | 15.7 | 21.6 | 8.9 | 98.7 | 15.5 |
|  | Khâi-Ma | 2.4 | 19.2 | 8.7 | 25.0 | 20.8 | 19.5 | 4.5 | 12.1 | 36.4 | 19.0 |
|  | Namaqua District | 12.2 | 17.6 | 8.6 | 23.3 | 31.5 | 18.2 | 31.0 | 11.4 | 83.3 | 17.6 |
| CSIRO 50 years | Richtersveld | 3.3 | 21.1 | 0.1 | 26.0 | 10.5 | 21.9 | 20.5 | 15.8 | 34.4 | 21.2 |
|  | Nama Khoi | 7.8 | 20.5 | 1.0 | 25.6 | 20.9 | 21.7 | 38.8 | 15.5 | 68.5 | 20.8 |
|  | Kamiesberg | 15.5 | 19.1 | 0.8 | 23.9 | 27.2 | 20.8 | 60.1 | 15.3 | 103.6 | 19.8 |
|  | Hantam | 12.6 | 19.4 | 7.1 | 25.5 | 35.8 | 20.1 | 39.3 | 13.4 | 94.7 | 19.6 |
|  | Karoo Hoogland | 13.5 | 17.3 | 13.4 | 24.0 | 37.7 | 18.0 | 20.3 | 11.3 | 84.9 | 17.6 |
|  | Khâi-Ma | 1.9 | 21.1 | 6.2 | 27.1 | 18.7 | 21.7 | 4.0 | 14.6 | 30.9 | 21.1 |
|  | Namaqua District | 10.4 | 19.3 | 6.5 | 25.2 | 29.1 | 20.2 | 30.7 | 13.7 | 76.6 | 19.6 |
| Miroc 50 years | Richtersveld | 2.4 | 21.4 | 0.1 | 26.3 | 6.1 | 21.8 | 17.5 | 15.6 | 26.1 | 21.3 |
|  | Nama Khoi | 6.4 | 20.8 | 1.4 | 25.7 | 13.1 | 21.6 | 34.7 | 15.1 | 55.6 | 20.8 |
|  | Kamiesberg | 11.4 | 19.4 | 1.2 | 24.3 | 17.1 | 20.8 | 56.9 | 15.0 | 86.5 | 19.9 |
|  | Hantam | 11.0 | 19.6 | 8.8 | 25.5 | 25.7 | 20.1 | 38.2 | 13.1 | 83.7 | 19.6 |
|  | Karoo Hoogland | 12.8 | 17.5 | 17.8 | 23.8 | 32.0 | 17.7 | 20.8 | 10.9 | 83.4 | 17.5 |
|  | Khâi-Ma | 1.6 | 21.3 | 8.1 | 27.0 | 13.5 | 21.6 | 4.3 | 14.2 | 27.5 | 21.0 |
|  | Namaqua District | 9.0 | 19.6 | 8.4 | 25.2 | 21.5 | 20.1 | 29.4 | 13.4 | 68.3 | 19.6 |
| Mpi 50 years | Richtersveld | 3.7 | 21.0 | 0.1 | 25.8 | 9.8 | 21.6 | 16.8 | 14.9 | 30.4 | 20.8 |
|  | Nama Khoi | 8.6 | 20.4 | 1.3 | 25.2 | 19.6 | 21.3 | 33.8 | 14.4 | 63.2 | 20.3 |
|  | Kamiesberg | 15.7 | 19.0 | 1.0 | 23.5 | 24.4 | 20.4 | 52.2 | 14.3 | 93.2 | 19.3 |
|  | Hantam | 13.8 | 19.1 | 8.7 | 24.9 | 34.6 | 19.8 | 36.7 | 12.3 | 93.7 | 19.0 |
|  | Karoo Hoogland | 14.1 | 17.1 | 16.5 | 23.4 | 39.1 | 17.5 | 21.2 | 10.2 | 90.9 | 17.0 |
|  | Khâi-Ma | 2.2 | 20.9 | 8.0 | 26.5 | 20.0 | 21.3 | 4.3 | 13.5 | 34.4 | 20.6 |
|  | Namaqua District | 11.1 | 19.1 | 8.0 | 24.7 | 28.7 | 19.8 | 28.3 | 12.6 | 76.1 | 19.1 |
| Average 50 years  (Models) | Richtersveld | 3.1 | 21.2 | 0.1 | 26.0 | 8.8 | 21.8 | 18.3 | 15.4 | 30.3 | 21.1 |
|  | Nama Khoi | 7.6 | 20.5 | 1.2 | 25.5 | 17.8 | 21.5 | 35.8 | 15.0 | 62.4 | 20.6 |
|  | Kamiesberg | 14.2 | 19.1 | 1.0 | 23.9 | 22.9 | 20.7 | 56.4 | 14.9 | 94.4 | 19.6 |
|  | Hantam | 12.4 | 19.4 | 8.2 | 25.3 | 32.0 | 20.0 | 38.0 | 12.9 | 90.7 | 19.4 |
|  | Karoo Hoogland | 13.5 | 17.3 | 15.9 | 23.7 | 36.3 | 17.7 | 20.8 | 10.8 | 86.4 | 17.4 |
|  | Khâi-Ma | 1.9 | 21.1 | 7.4 | 26.9 | 17.4 | 21.6 | 4.2 | 14.1 | 30.9 | 20.9 |
|  | Namaqua District | 10.1 | 19.3 | 7.7 | 25.0 | 26.4 | 20.1 | 29.5 | 13.2 | 73.7 | 19.4 |
| Average Change  (50 year - current) | Richtersveld | -0.7 | 1.5 | 0.0 | 1.4 | -2.0 | 1.5 | -1.4 | 1.6 | -4.1 | 1.5 |
|  | Nama Khoi | -1.6 | 1.7 | -0.2 | 1.6 | -3.2 | 1.7 | -2.4 | 1.8 | -7.3 | 1.7 |
|  | Kamiesberg | -3.1 | 1.6 | -0.1 | 1.6 | -4.6 | 1.6 | -2.4 | 1.7 | -10.3 | 1.6 |
|  | Hantam | -2.7 | 1.8 | -1.0 | 1.9 | -5.7 | 2.0 | -1.8 | 1.9 | -11.3 | 1.9 |
|  | Karoo Hoogland | -2.4 | 1.7 | -1.9 | 1.8 | -7.2 | 2.0 | -0.9 | 1.8 | -12.3 | 1.9 |
|  | Khâi-Ma | -0.5 | 1.9 | -1.2 | 1.9 | -3.5 | 2.0 | -0.3 | 2.0 | -5.4 | 2.0 |
|  | Namaqua District | -2.1 | 1.7 | -1.0 | 1.8 | -5.0 | 1.9 | -1.5 | 1.8 | -9.6 | 1.8 |

Table: Current and modelled seasonal and annual temperature and precipitation for the NDM and its local municipalities for the long term 2081-2100 time period

|  | Area | Spring Rain (mm) | Spring Temperature (°C) | Summer Rain (mm) | Summer Temperature (°C) | Autumn Rain (mm) | Autumn Temperature (°C) | Winter Rain (mm) | Winter Temperature (°C) | Annual Rain (mm) | Annual Temperature (°C) |
| --- | --- | --- | --- | --- | --- | --- | --- | --- | --- | --- | --- |
| Current Climate | Richtersveld | 3.8 | 19.7 | 0.1 | 24.7 | 10.8 | 20.3 | 19.7 | 13.8 | 34.4 | 19.6 |
|  | Nama Khoi | 9.2 | 18.9 | 1.4 | 23.9 | 21.0 | 19.8 | 38.2 | 13.2 | 69.8 | 19.0 |
|  | Kamiesberg | 17.3 | 17.6 | 1.1 | 22.3 | 27.5 | 19.1 | 58.8 | 13.2 | 104.8 | 18.0 |
|  | Hantam | 15.2 | 17.6 | 9.2 | 23.4 | 37.7 | 18.0 | 39.9 | 11.1 | 101.9 | 17.5 |
|  | Karoo Hoogland | 15.8 | 15.6 | 17.8 | 21.9 | 43.5 | 15.7 | 21.6 | 8.9 | 98.7 | 15.5 |
|  | Khâi-Ma | 2.4 | 19.2 | 8.7 | 25.0 | 20.8 | 19.5 | 4.5 | 12.1 | 36.4 | 19.0 |
|  | Namaqua District | 12.2 | 17.6 | 8.6 | 23.3 | 31.5 | 18.2 | 31.0 | 11.4 | 83.3 | 17.6 |
| CSIRO 100 years | Richtersveld | 2.4 | 22.7 | 0.1 | 27.9 | 12.4 | 23.9 | 17.9 | 17.4 | 32.8 | 23.0 |
|  | Nama Khoi | 6.2 | 22.2 | 1.2 | 27.7 | 25.5 | 23.9 | 35.4 | 17.2 | 68.3 | 22.8 |
|  | Kamiesberg | 12.6 | 20.8 | 1.0 | 26.0 | 32.5 | 22.9 | 52.9 | 17.0 | 99.1 | 21.7 |
|  | Hantam | 12.9 | 21.2 | 8.4 | 27.8 | 44.0 | 22.4 | 38.6 | 15.3 | 103.9 | 21.7 |
|  | Karoo Hoogland | 13.3 | 19.2 | 16.6 | 26.2 | 48.0 | 20.1 | 21.2 | 13.2 | 99.0 | 19.7 |
|  | Khâi-Ma | 1.9 | 23.0 | 7.6 | 29.4 | 25.4 | 24.1 | 4.3 | 16.6 | 39.2 | 23.3 |
|  | Namaqua District | 9.8 | 21.2 | 7.9 | 27.4 | 36.3 | 22.4 | 29.3 | 15.6 | 83.3 | 21.6 |
| Miroc 100 years | Richtersveld | 1.5 | 22.8 | 0.0 | 27.7 | 3.4 | 23.6 | 11.8 | 17.2 | 16.8 | 22.8 |
|  | Nama Khoi | 4.3 | 22.4 | 0.8 | 27.4 | 7.3 | 23.7 | 24.5 | 16.9 | 37.0 | 22.6 |
|  | Kamiesberg | 8.7 | 20.9 | 0.8 | 26.0 | 10.5 | 22.9 | 40.9 | 16.6 | 60.9 | 21.6 |
|  | Hantam | 7.5 | 21.5 | 6.3 | 27.7 | 16.3 | 22.7 | 27.6 | 14.9 | 57.8 | 21.7 |
|  | Karoo Hoogland | 8.9 | 19.3 | 13.8 | 25.7 | 22.9 | 20.3 | 16.1 | 12.6 | 61.7 | 19.5 |
|  | Khâi-Ma | 1.1 | 23.2 | 5.1 | 29.1 | 6.7 | 24.2 | 3.2 | 16.1 | 16.1 | 23.1 |
|  | Namaqua District | 6.2 | 21.3 | 6.2 | 27.1 | 13.9 | 22.5 | 21.3 | 15.1 | 47.7 | 21.5 |
| Mpi 100 years | Richtersveld | 4.0 | 22.4 | 0.0 | 27.3 | 5.3 | 23.2 | 13.1 | 17.2 | 22.4 | 22.5 |
|  | Nama Khoi | 9.7 | 22.0 | 0.8 | 27.2 | 10.7 | 23.3 | 28.3 | 17.1 | 49.5 | 22.4 |
|  | Kamiesberg | 17.8 | 20.5 | 0.6 | 25.4 | 13.4 | 22.2 | 42.0 | 16.7 | 73.8 | 21.2 |
|  | Hantam | 14.3 | 21.0 | 6.2 | 27.6 | 19.5 | 22.2 | 32.7 | 15.1 | 72.7 | 21.5 |
|  | Karoo Hoogland | 14.1 | 18.9 | 13.1 | 25.9 | 25.0 | 20.0 | 19.0 | 12.9 | 71.2 | 19.4 |
|  | Khâi-Ma | 2.4 | 22.7 | 4.9 | 29.1 | 10.2 | 23.8 | 3.7 | 16.5 | 21.2 | 23.0 |
|  | Namaqua District | 11.7 | 20.9 | 5.9 | 27.0 | 16.7 | 22.1 | 24.4 | 15.3 | 58.7 | 21.3 |
| Average 100 years  (Models) | Richtersveld | 2.6 | 22.6 | 0.0 | 27.6 | 7.1 | 23.6 | 14.3 | 17.2 | 24.0 | 22.8 |
|  | Nama Khoi | 6.8 | 22.2 | 0.9 | 27.4 | 14.5 | 23.6 | 29.4 | 17.1 | 51.6 | 22.6 |
|  | Kamiesberg | 13.0 | 20.7 | 0.8 | 25.8 | 18.8 | 22.7 | 45.3 | 16.8 | 77.9 | 21.5 |
|  | Hantam | 11.6 | 21.2 | 7.0 | 27.7 | 26.6 | 22.5 | 33.0 | 15.1 | 78.1 | 21.6 |
|  | Karoo Hoogland | 12.1 | 19.1 | 14.5 | 25.9 | 32.0 | 20.1 | 18.8 | 12.9 | 77.3 | 19.5 |
|  | Khâi-Ma | 1.8 | 22.9 | 5.9 | 29.2 | 14.1 | 24.0 | 3.7 | 16.4 | 25.5 | 23.1 |
|  | Namaqua District | 9.2 | 21.1 | 6.7 | 27.2 | 22.3 | 22.3 | 25.0 | 15.3 | 63.2 | 21.5 |
| Average Change  (100 year - current) | Richtersveld | -1.2 | 2.9 | 0.0 | 3.0 | -3.8 | 3.3 | -5.4 | 3.4 | -10.4 | 3.1 |
|  | Nama Khoi | -2.5 | 3.3 | -0.5 | 3.6 | -6.5 | 3.8 | -8.8 | 3.8 | -18.1 | 3.6 |
|  | Kamiesberg | -4.3 | 3.2 | -0.3 | 3.5 | -8.7 | 3.6 | -13.5 | 3.6 | -26.8 | 3.4 |
|  | Hantam | -3.6 | 3.7 | -2.2 | 4.2 | -11.1 | 4.4 | -6.9 | 4.1 | -23.8 | 4.1 |
|  | Karoo Hoogland | -3.7 | 3.6 | -3.3 | 4.1 | -11.5 | 4.4 | -2.9 | 3.9 | -21.4 | 4.0 |
|  | Khâi-Ma | -0.6 | 3.7 | -2.8 | 4.2 | -6.7 | 4.5 | -0.8 | 4.3 | -10.9 | 4.2 |
|  | Namaqua District | -3.0 | 3.5 | -1.9 | 3.9 | -9.2 | 4.2 | -6.0 | 3.9 | -20.0 | 3.9 |

| **SUMMER (DJF) Projected Temperature Change – Increase in °C CSIRO** | | **Legend** |
| --- | --- | --- |
| **50 Years** | **100 Years** |  |
| **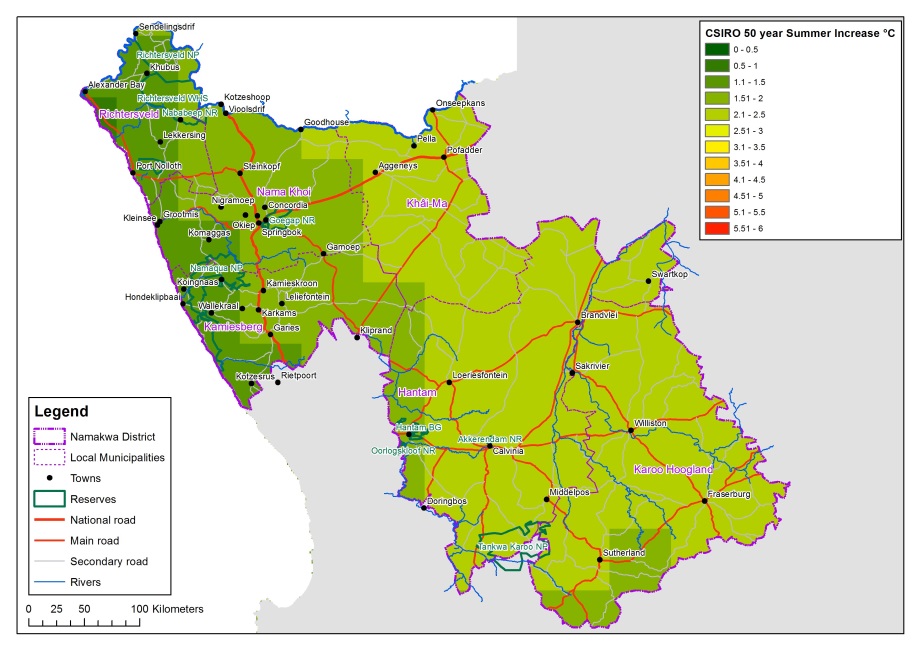** | **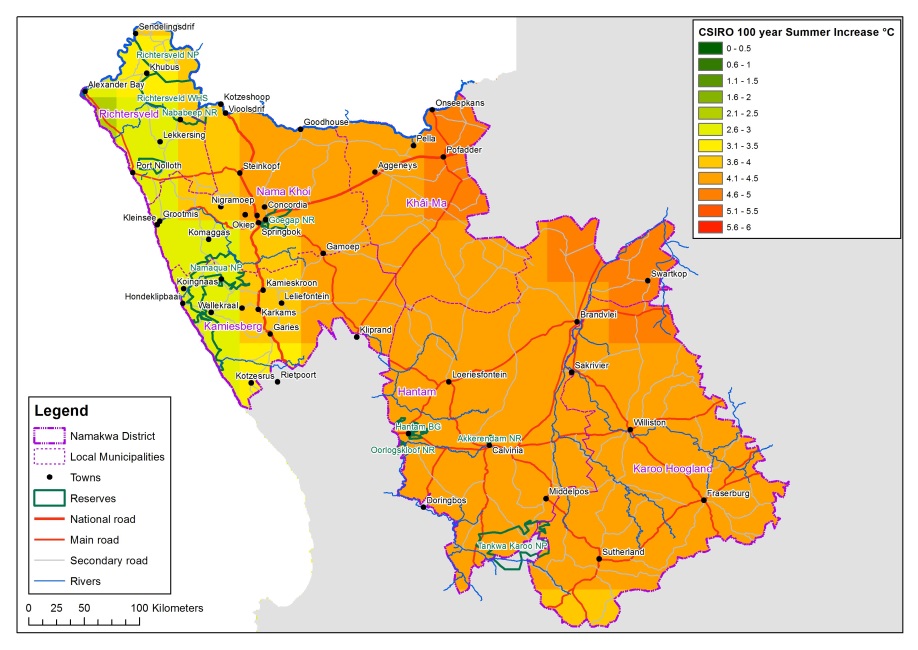** | 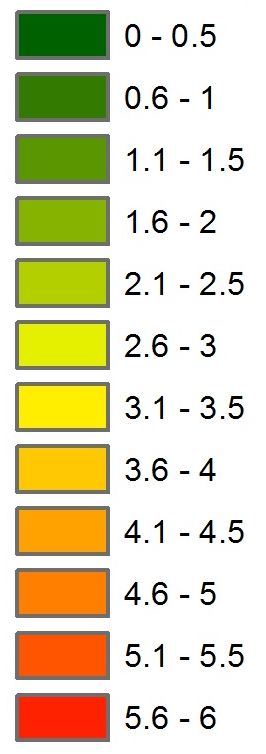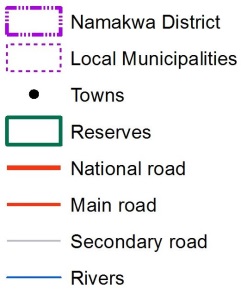 |
| **AUTUMN (MAM) Projected Temperature Change – Increase in °C CSIRO** | |  |
| **50 Years** | **100 years** |  |
| 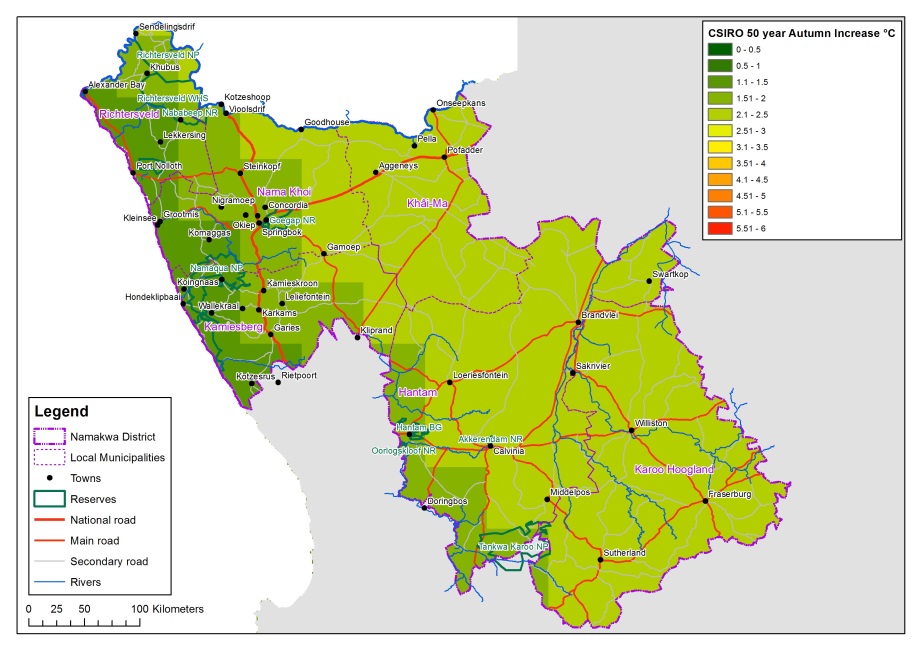 | 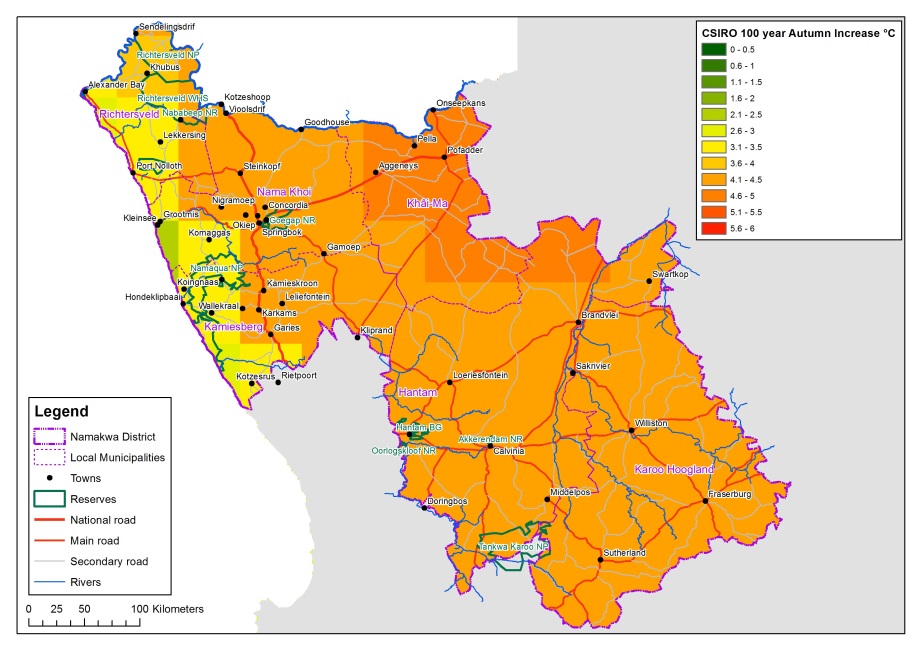 |  |

Figure: Summer and autumn season average temperature projection maps for the NDM, shown as increase in °C relative to current temperatures using the CSIRO model, clockwise from top left. 1) Medium term projected summer average temperatures, 2) long term projected summer average temperatures, 3) medium term projected autumn average temperatures, and 4) long term projected autumn average temperatures. Temperatures are projected to increase dramatically in the longer term in both summer and autumn.

| **AUTUMN (MAM) Rainfall % Change CSIRO** | | **Legend** |
| --- | --- | --- |
| **50 Years** | **100 Years** |  |
| 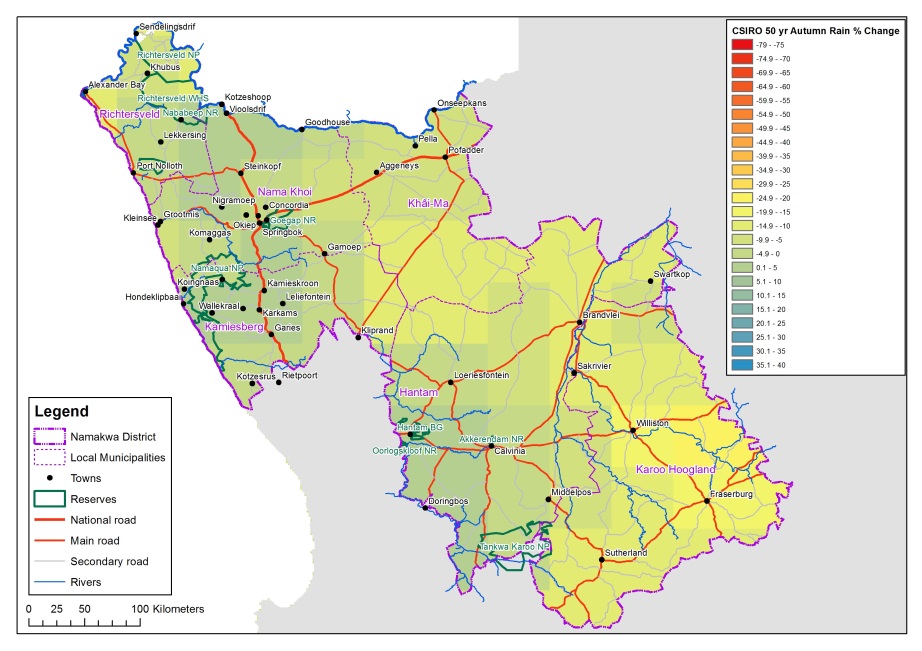 | 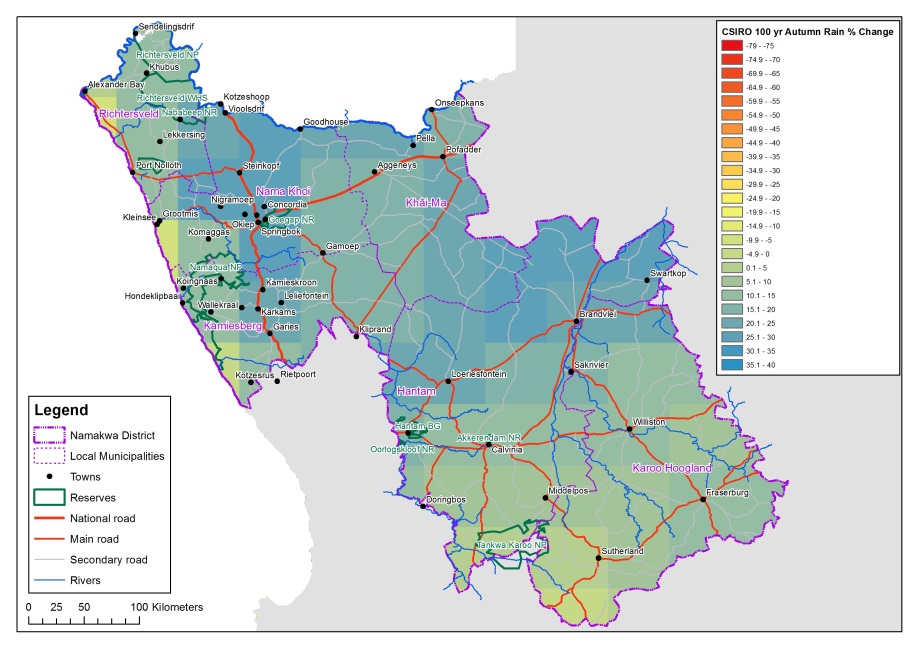 | 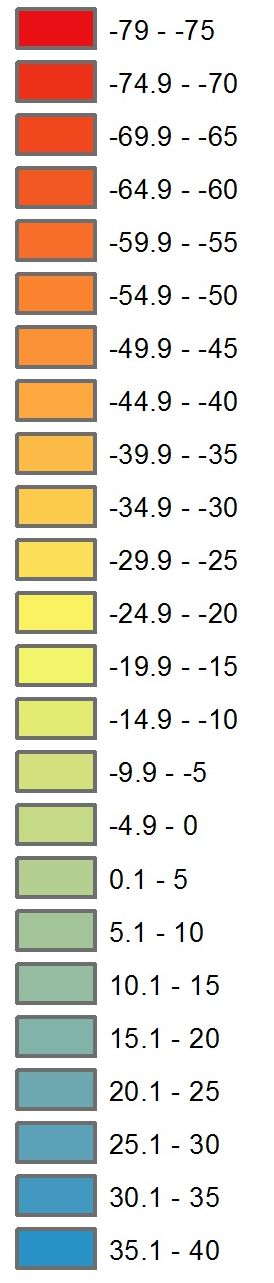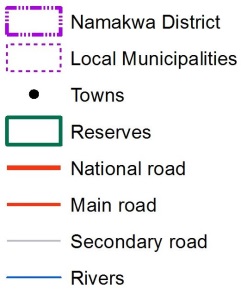 |
| **AUTUMN (MAM) Rainfall % Change MIROC** | |  |
| **50 Years** | **100 years** |  |
| 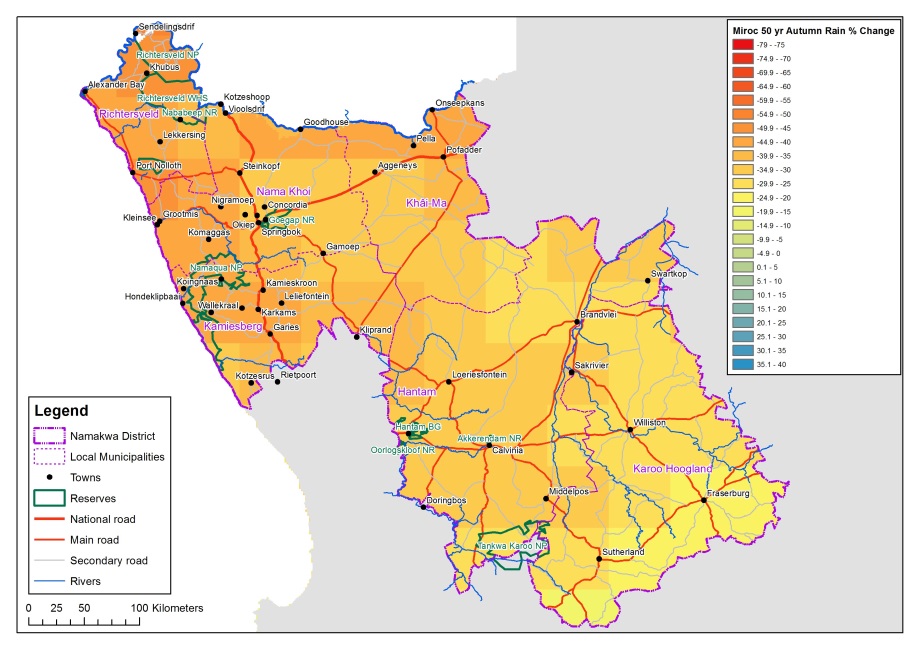 | 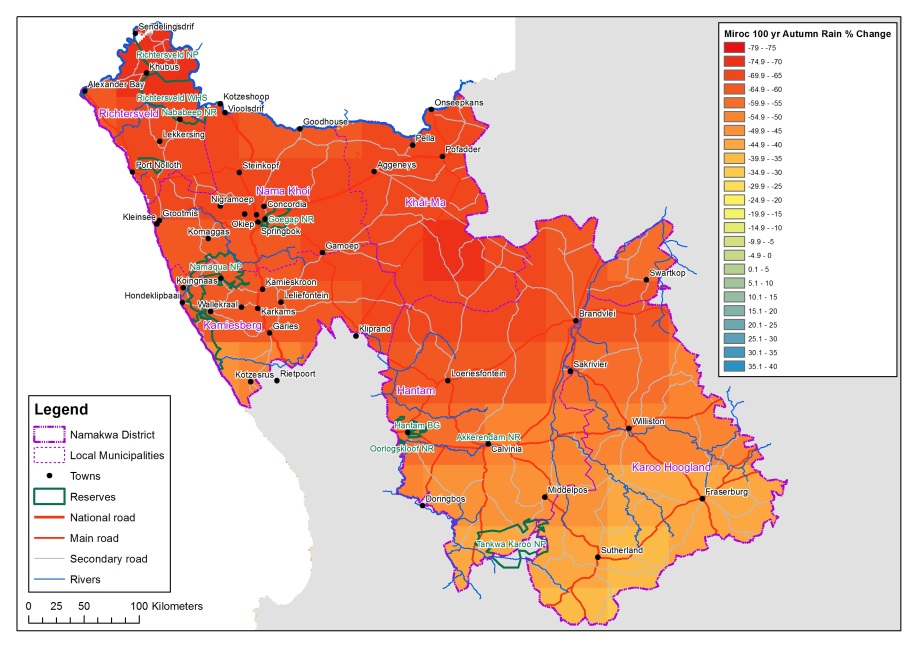 |  |

Figure: Autumn season rainfall change projection maps for the NDM, shown as a % change relative to current annual median rainfall comparing CSIRO and MIROC, clockwise from top left. 1) Medium term projected rainfall change in autumn CSIRO, 2) long term projected rainfall change in autumn CSIRO, 3) medium term projected rainfall change in autumn MIROC, and 4) long term projected rainfall change in autumn MIROC. The CSIRO model predicts long term wetting in the future for the NDM in the autumn months, while MIROC predicts severe drying.

Table: Current and modelled seasonal and annual temperature and precipitation for the ANDM and its local municipalities for the medium term 2040-2059 time period

Table: Current and modelled seasonal and annual temperature and precipitation for the ANDM and its local municipalities for the long term 2081-2100 time period

| **WINTER (JJA) Projected Temperature Change – Increase in °C CSIRO** | | **Legend** |
| --- | --- | --- |
| **50 Years** | **100 Years** |  |
| 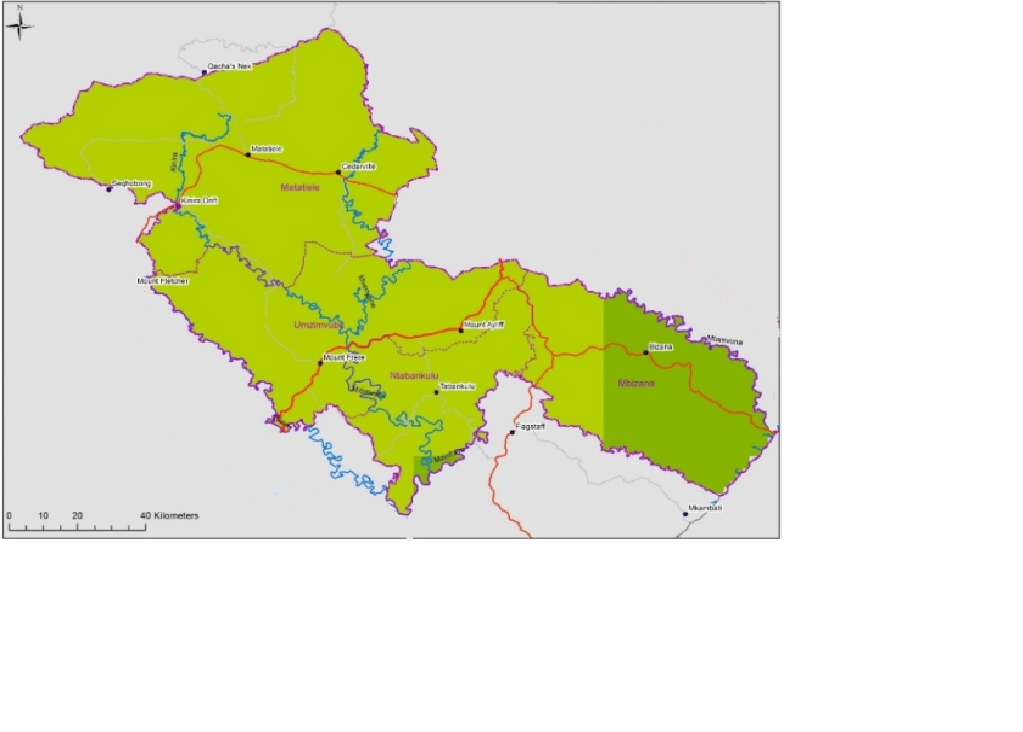 | 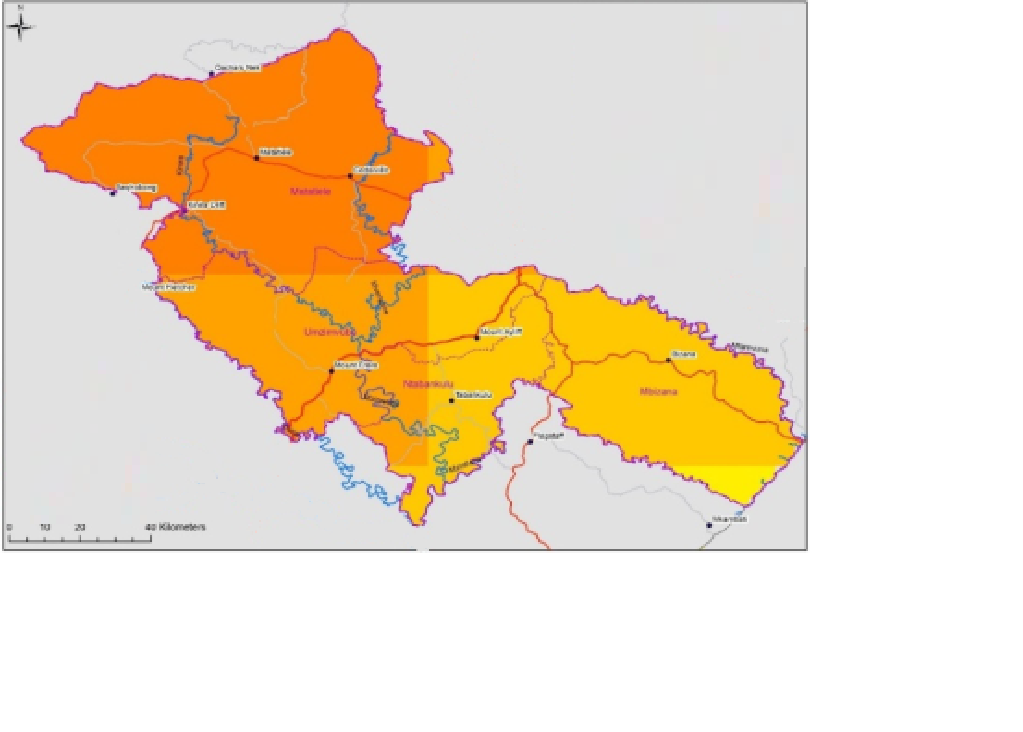 | 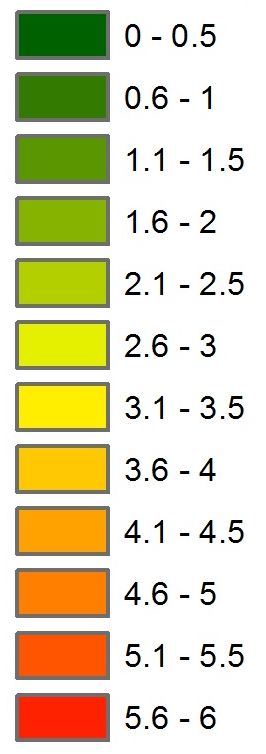 |
| **SPRING (SON) Projected Temperature Change – Increase in °C CSIRO** | |  |
| **50 Years** | **100 years** |  |
| 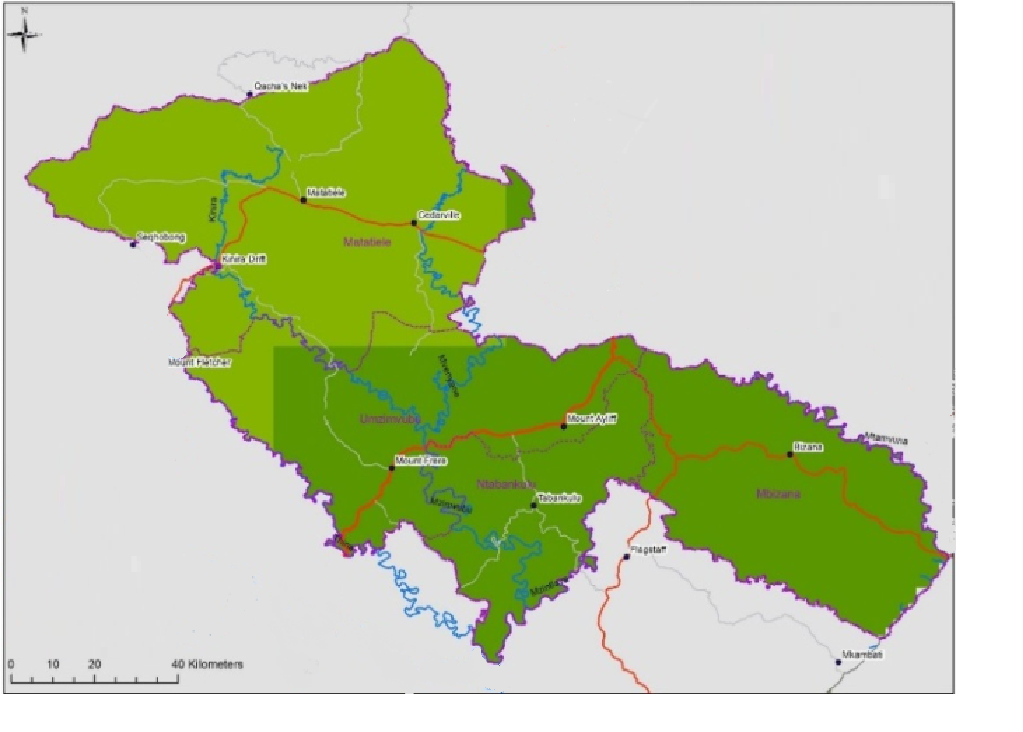 | 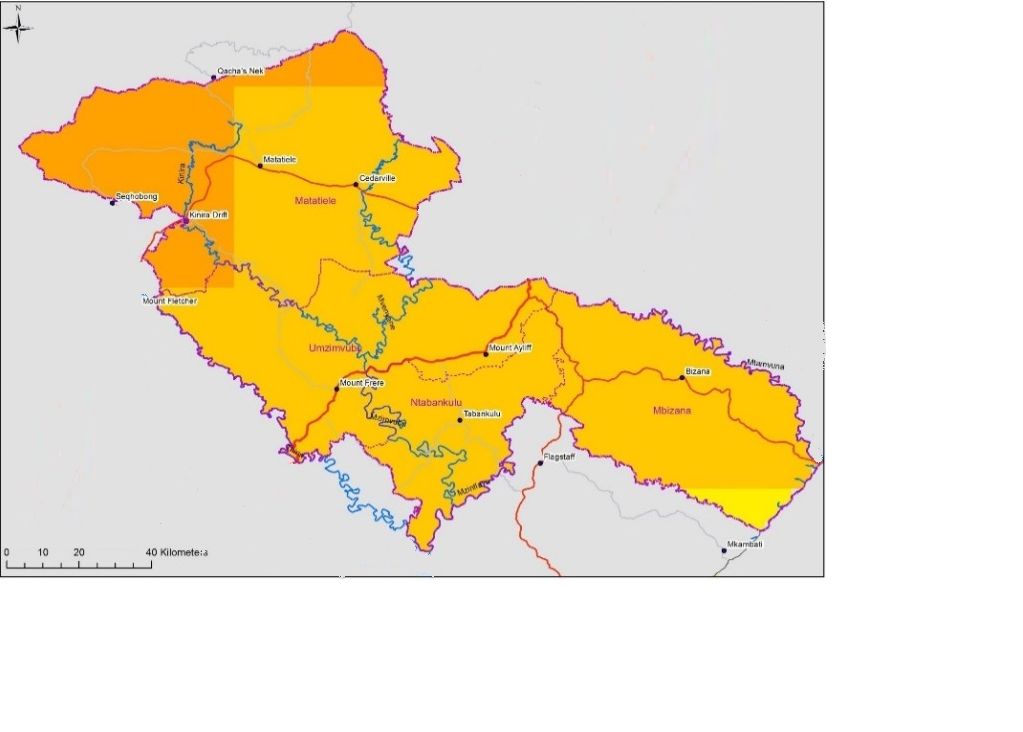 |  |

Supplementary Online Material: Winter and spring season average temperature projection maps for the ANDM, shown as increase in °C relative to current temperatures using the CSIRO model, clockwise from top left. 1) Medium term projected winter average temperatures, 2) long term projected winter average temperatures, 3) medium term projected spring average temperatures, and 4) long term projected spring average temperatures. Temperatures are projected to increase dramatically in the longer term in both summer and autumn.

| **SUMMER (DJF) Rainfall % Change CSIRO** | | **Legend** |
| --- | --- | --- |
| **50 Years** | **100 Years** |  |
| 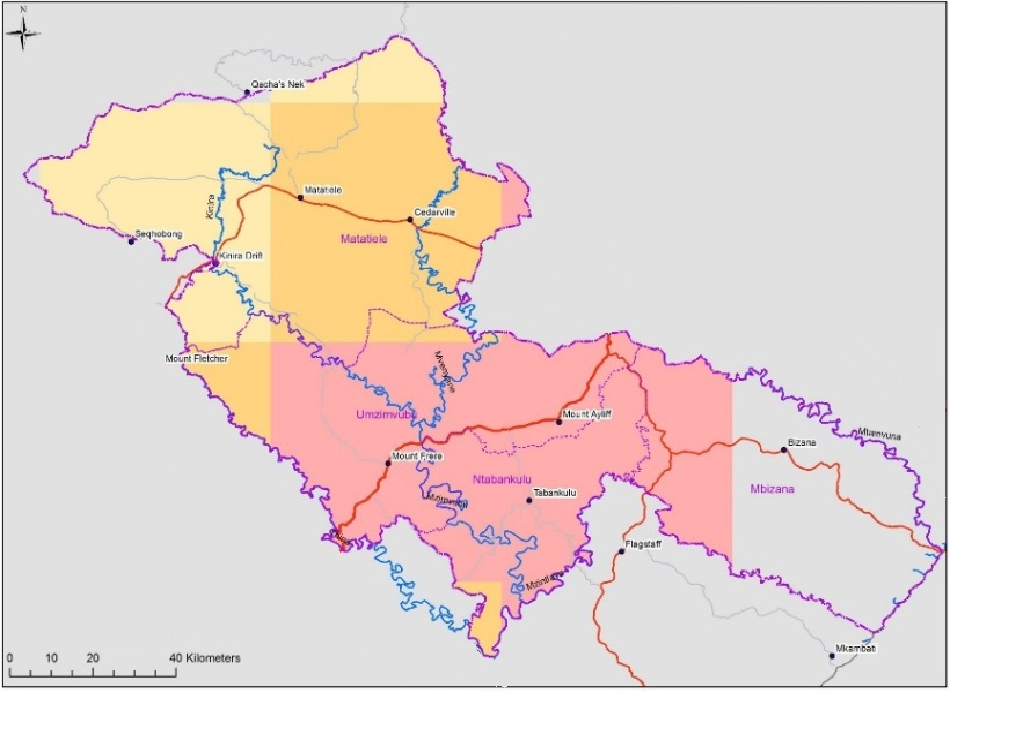 | 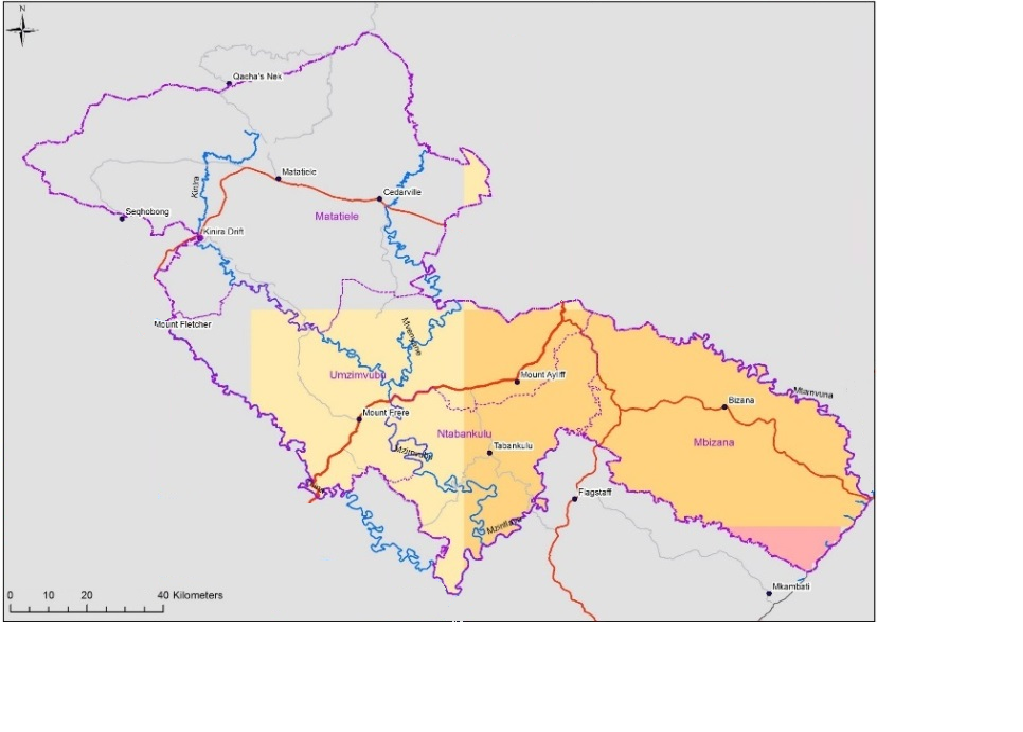 | 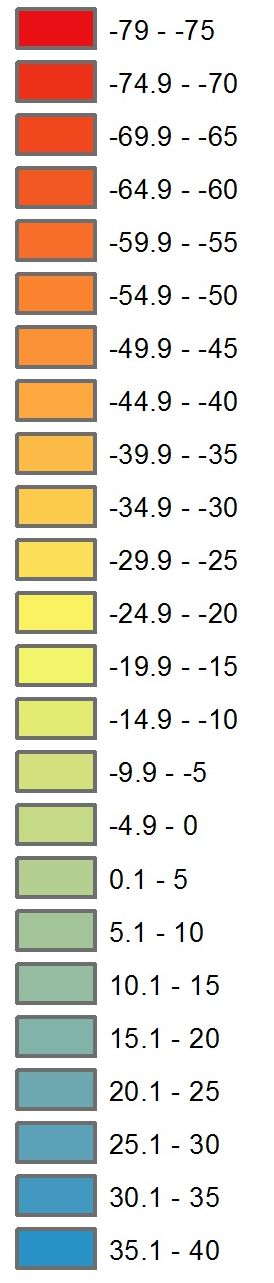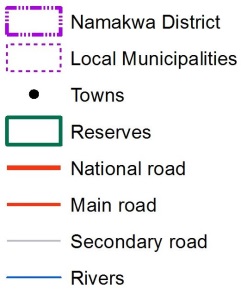 |
| **WINTER (JJA) Rainfall % Change CSIRO** | |  |
| **50 Years** | **100 years** |  |
| 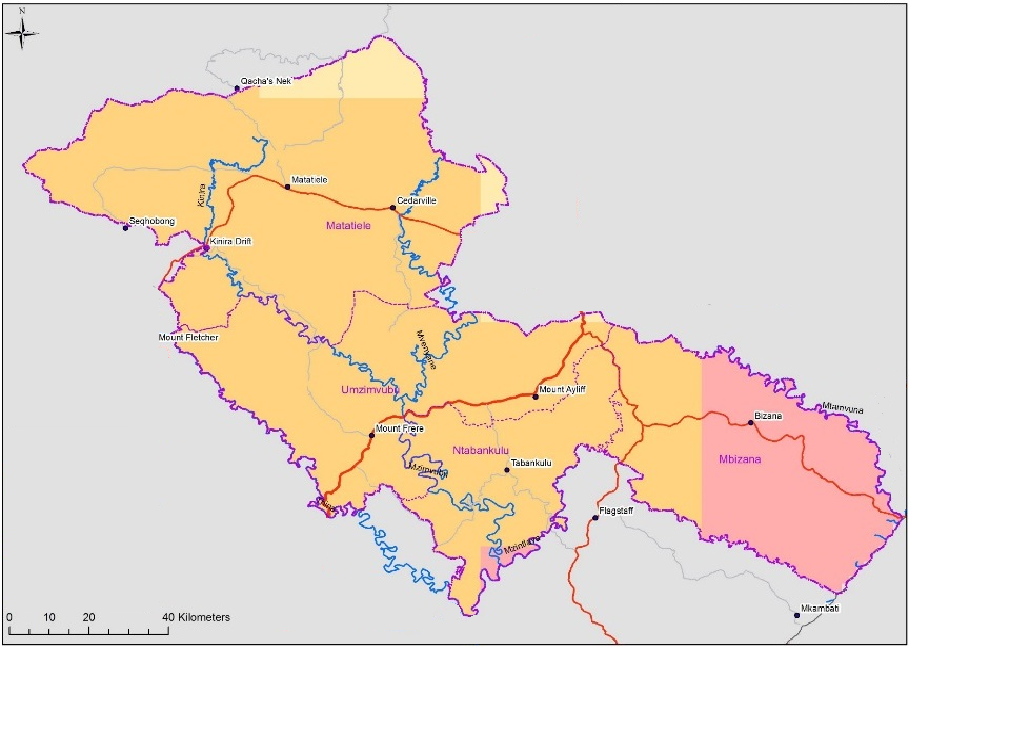 | 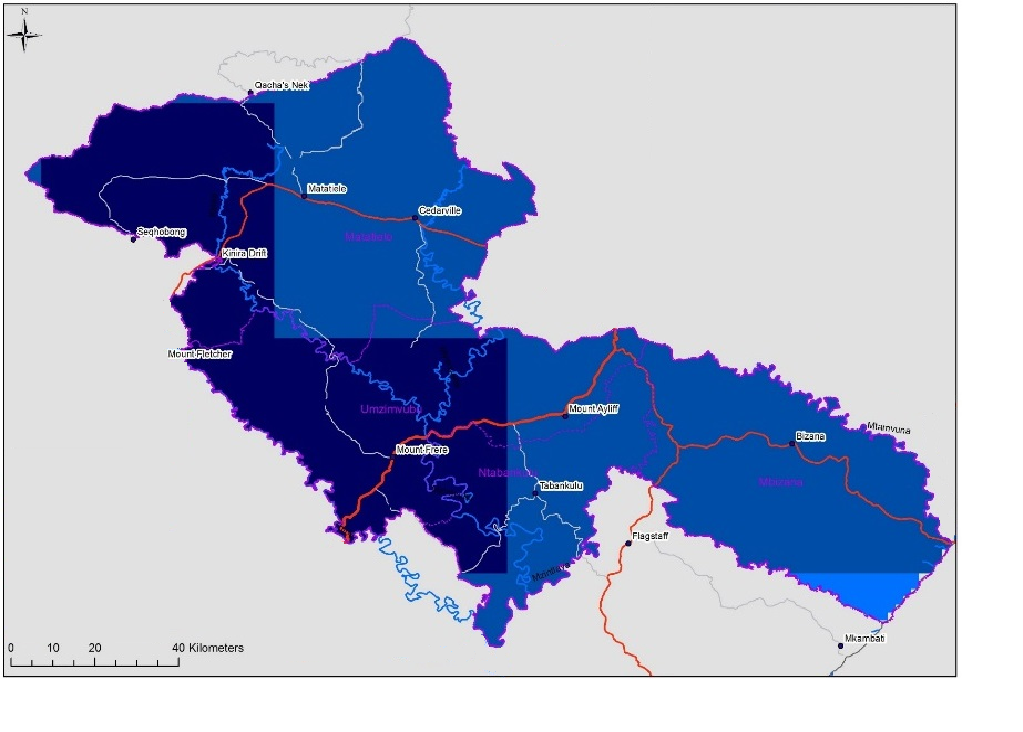 |  |

Supplementary Online Material: Summer and winter season rainfall change projection maps for the ANDM, shown as a % change relative to current annual median rainfall using the CSIRO model, clockwise from top left. 1) Medium term projected rainfall change in summer, 2) long term projected rainfall change in summer, 3) medium term projected rainfall change in winter, and 4) long term projected rainfall change in winter. The CSIRO model predicts long term wetting in the future for the ANDM in the winter months, and medium term drying in the summer months.
